# Lysosomal down-regulation of the mu opioid receptor is opposed by the Retromer complex

**DOI:** 10.1126/sciadv.adx8715

**Published:** 2026-03-20

**Authors:** Aleksandra Dagunts, Hayden Adoff, Brandon Novy, Lamya Ben Ameur, Monica De Maria, Arpiar Saunders, Braden T. Lobingier

**Affiliations:** ^1^ Department of Chemical Physiology and Biochemistry, Oregon Health and Science University, Portland, OR 97239, USA.; ^2^ Vollum Institute, Oregon Health and Science University, Portland, OR 97239, USA.

## Abstract

A critical homeostatic mechanism for regulating G protein–coupled receptor (GPCR) activity is agonist-induced GPCR endocytosis and trafficking to the lysosome for proteolytic down-regulation. The mu opioid receptor (MOR) is a notable example of this type of cellular regulation, where prolonged exposure to high-efficacy opioid drugs causes MOR to traffic to the lysosome. Here, we used functional genomics to identify cellular proteins that control MOR lysosomal down-regulation. We found that the central regulator of MOR postendocytic trafficking is the Retromer complex, which rescues MOR from opioid-induced down-regulation by promoting MOR recycling from endosomes to the plasma membrane. Critically, MOR accesses the Retromer recycling pathway through its noncanonical bileucine recycling motif, and this mechanism controls how MOR is regulated following chronic exposure to opioid drugs. Additionally, we show that this bileucine pathway for Retromer-based recycling is present in other classes of membrane proteins including the glucose transporter GLUT4.

## Teaser

The Retromer complex rescues mu opioid receptors from lysosomal down-regulation after exposure to high-efficacy opioids.

## INTRODUCTION

G protein–coupled receptor (GPCR) signaling is responsible for the physiological responses to many hormones and neurotransmitters, and regulation of GPCR function is required for cellular homeostasis (*1*). A critical mechanism for GPCR regulation involves agonist-induced receptor trafficking (*2*). Agonist-bound GPCRs are endocytosed from the plasma membrane and trafficked to endosomes where they are sorted to lysosomes for proteolytic down-regulation (hereafter called lysosomal down-regulation). Consequently, prolonged or repeated agonist stimulation can cause a loss of cellular responsiveness to agonist when receptor down-regulation at lysosomes outpaces new receptor synthesis (*3*, *4*). However, some types of GPCRs can resist lysosomal down-regulation following agonist-induced endocytosis. These receptors contain specific sequences of amino acids called recycling motifs in their cytoplasmic-facing C-terminal tails that can be recognized by endosomal recycling complexes (*5*, *6*). Endosomal recycling complexes sort GPCRs with recycling motifs into endosomal tubules for return to the plasma membrane, thereby preventing GPCRs from reaching the lysosome (*7*). Thus, endosomal recycling of GPCRs acts as a critical checkpoint to determine the postendocytic fate of receptors by selecting specific GPCRs to be rescued out of the endosomal-lysosomal pathway. Overall, endosomal recycling acts to slow agonist-dependent GPCR lysosomal down-regulation and preserves cellular responsiveness to agonists (*7*–*9*).

Despite the importance of endosomal recycling in GPCR regulation, the mechanisms controlling this pathway remain incompletely understood. The best-characterized GPCR endosomal recycling pathway functions through sorting nexin 27 (SNX27). SNX27 recognizes and binds GPCRs with class I PDZ binding motifs ([D/E][S/T]xΦ-COOH, where Φ is hydrophobic and x can be any amino acid) in their C-terminal tail, and links the receptor to additional recycling complexes (*10*–*13*). Either loss of SNX27 or mutation of the PDZ binding motif in the receptor increases the rate of agonist-induced GPCR down-regulation, underscoring the role of endosomal recycling in protecting GPCRs from delivery to lysosomes (*10*). However, only about 4% of GPCRs contain a class I PDZ binding motif (*5*, *14*), and some GPCRs have been shown to recycle using motifs that do not match any known consensus recycling motif (*15*–*17*), suggesting the existence of additional mechanisms by which GPCRs can escape lysosomal down-regulation through endosomal recycling.

One example of a GPCR with a noncanonical recycling motif is the mu opioid receptor (MOR). MOR mediates the physiological effects of many opioid drugs, and endocytosis and postendocytic trafficking of MOR have been implicated in the development of pharmacological tolerance in response to high-efficacy opioids (*3*, *18*–*20*). Prior work identified a novel type of recycling motif in the final 17 amino acids of the MOR C-terminal tail, defined by the sequence LENL, that was necessary and sufficient to protect opioid receptors (ORs) from agonist-induced lysosomal down-regulation by promoting OR recycling from endosomes (*21*). Extensive mutational analysis defined a bileucine core (LxxL) to the LENL motif and showed that both leucines, but not the intervening or surrounding residues, were critical for recycling (*21*). However, the LENL sequence does not match any consensus motif recognized by endosomal recycling complexes (fig. S1A) (*22*). Thus, the mechanism by which LENL can induce MOR recycling and thus slow its lysosomal down-regulation is unknown (*7*, *23*).

We recently developed a chemical biology and functional genomics platform for unbiased identification of proteins involved in lysosomal down-regulation of GPCRs (*24*). This approach is based on a highly sensitive fluorogenic biosensor for GPCR expression that uses a genetic fusion between a GPCR and the engineered ascorbate peroxidase (APEX2) (*25*). GPCR-APEX2 can be used to measure GPCR expression in cells by quantifying the APEX2-dependent activation of the fluorogenic substrate AMPLEX UltraRed (AUR). The ability of APEX2 to activate AUR is quenched if APEX2 is delivered to the lumen of lysosomes (*24*). Consequently, agonist-induced trafficking of GPCR-APEX2 to the lysosome results in reduced AUR activation and loss of fluorescence. An advantage of this method is that it is compatible with pooled genetic screens, allowing for genome-wide interrogation using approaches like CRISPR interference (CRISPRi) to identify novel genetic regulators of GPCR expression throughout the receptor life cycle including in the secretory, endocytic, and endosomal-lysosomal pathways (*24*).

Here, we took a functional genomics approach and used our GPCR-APEX2/AUR biosensor to identify what cellular proteins regulate opioid-induced down-regulation of MOR. Our screen identified multiple genes regulating OR down-regulation through the endosomal-lysosomal pathway including all three subunits of the endosomal recycling complex Retromer. Leveraging this genetic finding, we showed that the bileucine motif LENL is a novel class of recycling motifs that give access to the Retromer-based endosomal recycling pathway. We found that Retromer functions with the LENL recycling motif to protect MOR from opioid-induced down-regulation by promoting MOR recycling from endosomes and that this process is conserved across multiple human cell types and primary rodent striatal neurons in vitro. Last, using a custom bioinformatics pipeline, we found that the bileucine LxxL core of the LENL recycling motif is present in the C-terminus of a number of membrane proteins in the human proteome, suggesting the potential for this to be a broadly used mechanism in membrane protein trafficking. Selecting the glucose transporter GLUT4 as an example, we demonstrated that the bileucine sequence in the GLUT4 C-terminal tail (LEYL) is a bona fide recycling motif that is both necessary and sufficient for postendocytic entry into the Retromer-recycling pathway. Together, our study identified a mechanism by which membrane proteins with bileucine recycling motifs can enter the endosomal Retromer pathway and demonstrated that this mechanism is critical to protect ORs from lysosomal down-regulation following prolonged exposure to high-efficacy opioids.

## RESULTS

### The GPCR-APEX2/AUR down-regulation assay captures changes in GPCR endosomal recycling

To understand the cellular factors that control opioid-induced lysosomal down-regulation of MOR, we wanted to take a functional genomics approach with a focus on identifying the genes that function with the noncanonical bileucine motif, LENL, to promote MOR endosomal recycling and thus oppose MOR lysosomal down-regulation. We had recently developed a high-sensitivity fluorogenic biosensor for GPCR expression and down-regulation in cells, GPCR-APEX2/AUR (*24*). In our previous study, we applied this approach to identify genes that promote agonist-dependent GPCR down-regulation using the delta opioid receptor (DOR) as a model nonrecycling GPCR, but we reasoned that our method could also capture the inverse process: genes that oppose GPCR lysosomal down-regulation by promoting endosomal recycling.

Several steps were required to successfully adapt our GPCR-APEX2/AUR functional genomics pipeline. First, we needed to ensure that changes in MOR down-regulation caused by loss of LENL function could be reliably detected by the GPCR-APEX2/AUR sensor. Second, we needed a strategy to narrow in on the subset of genes that function specifically with the LENL recycling motif in the context of a genome-wide CRISPRi screen capable of finding hits involved in the entire life cycle of a GPCR.

**Fig. 1.**
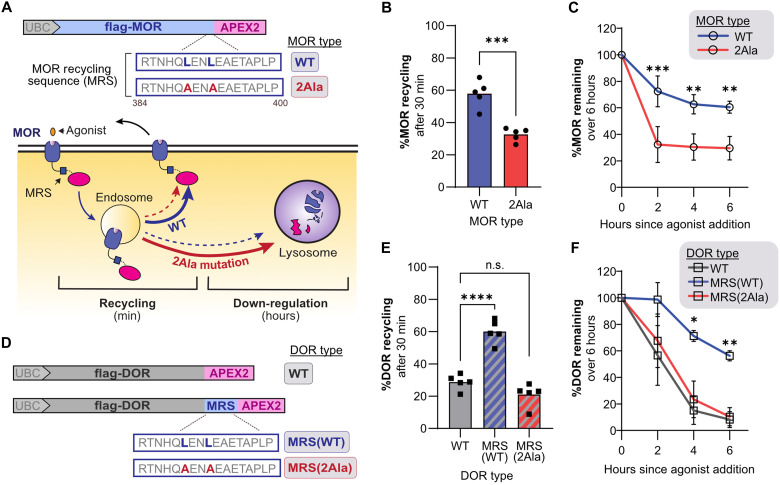
The GPCR-APEX2 down-regulation assay captures changes in GPCR endosomal recycling. (**A**) Construct design of APEX2-tagged MOR(WT) and MOR(2Ala) driven by the ubiquitin C (UBC) promoter, and schematic depicting their expected cellular trafficking over time, including major (solid) and minor (dotted) pathways. (**B**) Percent recycling of internalized MORs in stably expressing HEK293-FLP cells following 30 min of treatment with 10 μM DAMGO followed by 30 min of treatment with 10 μM naloxone (*n* = 5; two-tailed paired *t* test, *P* = 0.00040). (**C**) Percent MOR remaining in stably expressing HEK293-FLP cells following treatment with 10 μM DAMGO for 0, 2, 4, or 6 hours normalized to no agonist treatment [*n* = 3; two-way repeated measures (RM) analysis of variance (ANOVA) with Šidák’s multiple comparisons correction: *P* = 0.020 for receptor type effects; *P* < 0.0001 for time effects; *P* = 0.0008, 0.0025, and 0.0031 for MOR(WT) versus MOR(2Ala) at 2, 4, and 6 hours, respectively]. (**D**) Construct design of APEX2-tagged DOR(WT), DOR-MRS(WT), and DOR-MRS(2Ala). (**E**) Percent recycling of internalized DORs in stably expressing HEK293-FLP cells following 30 min of treatment with 10 μM DADLE followed by 30 min of treatment with 10 μM naloxone in HEK293-FLP cells [*n* = 5; one-way RM ANOVA with Dunnett’s multiple comparisons correction: *P* < 0.0001 for DOR(WT) versus DOR-MRS(WT); *P* = 0.19 for DOR(WT) versus DOR-MRS(2Ala)]. (**F**) Percent DOR remaining in stably expressing HEK293-FLP cells following treatment with 10 μM DADLE for 0, 2, 4, or 6 hours in HEK293-FLP cells normalized to no agonist treatment [*n* = 3; two-way RM ANOVA with Dunnett’s multiple comparisons correction: *P* = 0.0120 for receptor type effects; *P* = 0.0043 for time effects; *P* = 0.25, 0.016, and 0.0011 for DOR(WT) versus DOR-MRS(WT) at 2, 4, and 6 hours, respectively; *P* = 0.58, 0.37, and 0.66 for DOR(WT) versus DOR-MRS(2Ala) at 2, 4, and 6 hours respectively]. WT, wild type; n.s., not significant.

To address the first goal, we developed a model cell line to study MOR trafficking: human embryonic kidney 293-Flp-In cells (HEK293-FLP) that stably express MOR_WT_ (flag-MOR_WT_-APEX2) or the MOR mutant, MOR_2Ala_, in which the two essential leucines of the LENL motif were mutated to alanines (flag-MOR_2Ala_-APEX2), under the low-expressing ubiquitin C (*UBC*) promoter (Fig. 1A). To establish general function of the APEX2-tagged receptors, we assessed MOR expression as well as signaling in response to the opioid peptide agonist DAMGO. We found that both MOR_WT_ and MOR_2Ala_ expressed and showed agonist-dependent signaling, which is consistent with previous observations that MOR tolerates a C-terminal APEX2 tag (fig. S1, B to D) (*26*, *27*).

We next sought to verify that the APEX2 tag did not disrupt MOR trafficking. We first measured loss of MOR from the cell surface after 30 min of DAMGO treatment using field-standard flow cytometry assays (*21*, *23*, *28*, *29*). Under these conditions, the amount of cell surface receptor is primarily set by the rate of endocytosis (which removes receptors from the cell surface), but recycling also makes a small but measurable contribution during this period by returning receptors to the cell surface (*10*, *21*, *30*). We measured that 50% (±4.0%) of MOR_WT_ and 58% (±3.1%) of MOR_2Ala_ receptors were lost from the cell surface (i.e., internalized) following DAMGO treatment, consistent with robust agonist-dependent endocytosis modulated by loss of recycling in the MOR_2Ala_ condition (fig. S1, C and E). To directly assess MOR recycling, we quantified changes in surface MOR expression following sequential agonist and antagonist treatments that induce and then prevent receptor endocytosis, respectively (*21*, *23*, *28*, *29*). We found a ~50% reduction in percent recycling between MOR_WT_ (58 ± 8.3%) and MOR_2Ala_ (32.58 ± 4.0%), which is a similar effect size to previous studies of MOR, suggesting that LENL-based recycling was not perturbed by the APEX2 tag (Fig. 1B) (*21*). The residual recycling that we and others have measured in MORs lacking a functional LENL motif is consistent with the observation that GPCRs can traffic, albeit inefficiently, through a general “bulk flow” recycling pathway that is not dependent on recycling motifs (*7*, *21*, *31*–*33*). Having verified that the APEX2 tag did not inhibit MOR signaling or trafficking, we next sought to determine whether our GPCR-APEX2/AUR assay could capture the increased rate in delivery to the lysosome that occurs upon inhibition of GPCR recycling. We used the GPCR-APEX2/AUR assay to measure the rate of MOR lysosomal delivery over 6 hours of agonist treatment and found that MOR_WT_ showed a small amount of down-regulation that was greatly increased in the MOR_2Ala_ mutant (Fig. 1C). These results demonstrate that the GPCR-APEX2/AUR assay could accurately capture the enhanced rate of lysosomal down-regulation resulting from inhibition of MOR recycling.

Our second goal was to develop a functional genomics strategy that would allow us to reliably filter our genetic hits for candidate genes specifically responding to the LENL motif. We thought that adding a filtering step to our functional genomics pipeline would be important for our approach because, as we had observed before, there are potentially hundreds of genetic modulators of GPCR expression and trafficking acting across the entire GPCR life cycle (*24*). To solve this challenge, we decided on a screen design where we compared two highly related GPCRs that differed in one specific feature: the presence of the LENL recycling motif. We reasoned that these two GPCRs would share genes that affected expression, secretory trafficking, and endocytosis but differ in those genes that function with and downstream of the LENL motif in postendocytic GPCR sorting. In selecting what to compare, we leveraged two points: (i) Our previous genome-wide CRISPRi screen using the GPCR-APEX2/AUR assay was focused on the DOR, a poorly recycling GPCR without a recycling motif (*21*, *34*, *35*); and (ii) recycling motifs are modular and transferable, and, thus, pathway-specific recycling can be conferred to a poorly-recycling GPCR by genetically adding a recycling motif. Thus, we reasoned that we could leverage our already completed DOR dataset by conducting a second screen on DOR with an appended MOR recycling sequence (MRS) (*24*).

To verify that grafting the LENL recycling motif onto DOR would induce sequence-specific recycling in the presence of the APEX2 tag, we created HEK293-FLP cells that stably expressed either DOR_WT_ (flag-DOR_WT_-APEX2) or DOR tagged at its C terminus with the final 17 amino acids of MOR (MRS) containing either the functional [flag-DOR_MRS(WT)_-APEX2] or mutated [flag-DOR_MRS(2Ala)_-APEX2] LENL recycling motif (Fig. 1D). All three APEX2-tagged DOR constructs expressed, signaled, and underwent agonist-dependent endocytosis (fig. S1, F to H). We next examined DOR recycling following stimulation with the opioid peptide DADLE and found that DOR with a functional recycling motif, but not a mutated motif, showed enhanced recycling (Fig. 1E and fig. S1F). Last, we examined whether DOR_MRS(WT)_ would be a good candidate to capture the effects of recycling on GPCR lysosomal down-regulation. We successfully captured the rerouting of DOR_MRS(WT)_ away from the lysosome using the GPCR-APEX2/AUR down-regulation sensor, which measured fivefold more DOR remaining in cells expressing DOR_MRS(WT)_ over 6 hours of agonist stimulation (Fig. 1F). Together, these results confirmed the suitability of DOR_MRS(WT)_ for use in a screen to identify LENL-specific trafficking interactors using the GPCR-APEX2/AUR approach.

Last, an additional useful aspect of the DOR chimera studies is that they allowed us to examine whether GPCR surface expression level or degree of agonist-induced endocytosis strongly shaped postendocytic trafficking. Specifically, we observed that, although DOR expresses at higher levels than MOR (fig. S1, I and J) and undergoes a greater amount agonist-induced endocytosis (fig. S1, E and H), the amount of LENL-dependent recycling that occurs is very similar to MOR (Fig. 1, B and E). This observation suggests that, in our cell models, the postendocytic fate of ORs is primarily controlled by the presence or absence of a recycling motif and is not rate limited by the endosomal sorting machinery.

### The LENL motif requires Retromer to oppose agonist-induced OR down-regulation

**Fig. 2.**
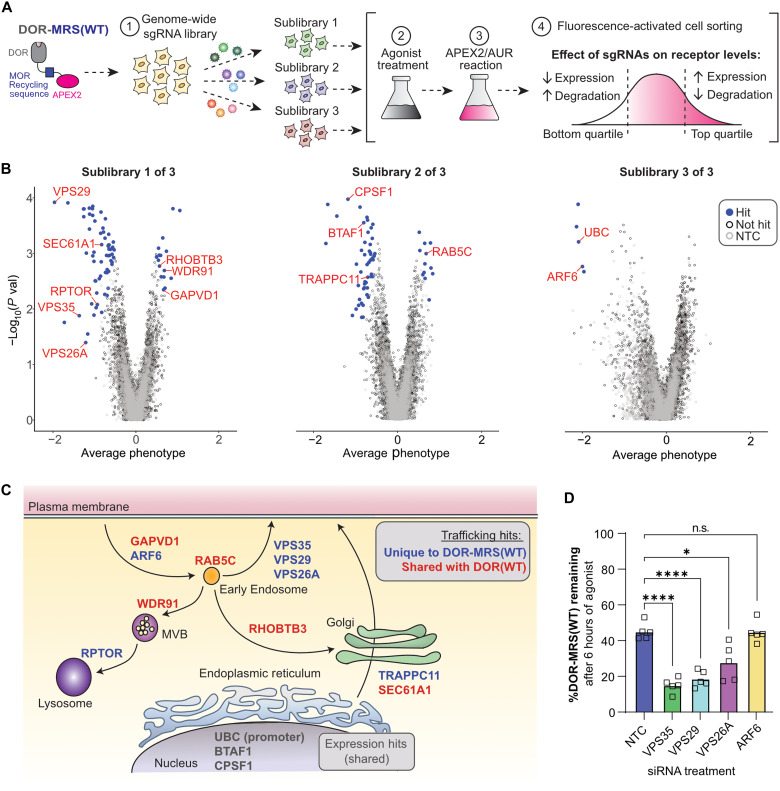
The LENL motif requires Retromer to oppose agonist-induced OR down-regulation. (**A**) Screen design schematic. (**B**) Volcano plots of relative gene enrichment in HEK293-FLP cells stably expressing DOR-MRS(WT) sorted into bottom and top fluorescence quartiles following the APEX2/AUR reaction, divided by sublibrary. Relative sgRNA enrichment between populations was analyzed with a Mann-Whitney *U* test from an *n* = 1 independent experiment. sgRNAs with a false discovery rate of <0.05 are denoted as hits (blue circles), nontargeting control (NTC) sgRNAs are depicted as open circles, and all other genes are depicted as gray circles. Select hits involved in receptor expression and trafficking are annotated in red. (**C**) Cartoon showing proposed location of action for hits involved in receptor expression and trafficking. Trafficking hits that were also found in our previous DOR(WT) screen are depicted in red, while hits that were unique to DOR-MRS(WT) are depicted in blue. Select hits proposed to be involved in gene expression are depicted in gray. (**D**) Percent DOR-MRS(WT) remaining in stably expressing HEK293-FLP cells following siRNA knockdown of select hits treated with 6 hours of 10 μM DADLE followed by the GPCR-APEX2/AUR reaction, normalized to no agonist treatment (*n* = 5; one-way RM ANOVA with Dunnett’s multiple comparisons correction: *P* < 0.0001, < 0.0001, = 0.011, and > 0.99 for NTC versus VPS35, VPS29, VPS26A, and ARF6, respectively). n.s., not significant.

Our functional genomics strategy was to compare the results from our prior genome-wide CRISPRi screen using the GPCR-APEX2/AUR with DOR_WT_ to a new genome-wide CRISPRi screen on DOR_MRS(WT)_ (*24*). To perform this new screen, we used the same approach as in our previous study and created a reporter line that stably expressed both dCas9-Krab and DOR_MRS(WT)_-APEX2 in HEK293-FLP cells. We then divided the genome-wide CRISPRi library into three sublibraries and transduced the reporter cell line with each sublibrary for an ~300-fold single guide RNA (sgRNA) coverage. Following 8 days of gene knockdown, cells were stimulated with agonist to induce receptor endocytosis and the amount of remaining DOR_MRS(WT)_ in individual cells was determined with the GPCR-APEX2/AUR assay. Cells were then sorted into the top and bottom quartiles on the basis of their individual fluorescence to identify sgRNAs that decreased GPCR expression (enriched in bottom quartile) or sgRNAs that increased GPCR expression (enriched in top quartile) after agonist exposure (Fig. 2A). We hypothesized that genes potentially involved in LENL-based recycling would be enriched in the bottom quartile because their loss would lead to a decrease in GPCR recycling, a subsequent increase in lysosomal down-regulation, and loss of overall expression in the cell.

Next-generation sequencing revealed that five of the five sgRNAs were found for 88.82% of every gene (and four of five were found for 99.02%), suggesting no large bottle necks in the workflow. The screen identified 146 hits (Fig. 2B and table S1). Consistent with our previous findings, analyzing only the data from the genome-wide screen on DOR_MRS(WT)_ revealed genes linked to membrane protein expression, secretory and endocytic trafficking, as well as the internal positive control: sgRNAs that target *UBC*, the promoter driving DOR_MRS(WT)_ expression (Fig. 2C) (*24*). Most of these hits (81.5%) from our model HEK293 cell line are also expressed in multiple types of MOR-expressing mouse neurons, demonstrating broad conservation of genes for GPCR expression and trafficking (table S2).

We then sought to identify candidate genes that might function with the bileucine LENL motif to oppose agonist-induced GPCR lysosomal down-regulation. To do this, we first filtered hits for those whose loss promoted DOR_MRS(WT)_ down-regulation (found on left side of volcano plots). From this list, we identified hits that were only found in the DOR_MRS(WT)_ screen and not the DOR_WT_ screen (Fig. 2C and table S1). Several genes had known roles in endosomal trafficking, including *VPS35*, *VPS29*, *VPS26A*, and *ARF6*. Three of these genes—*VPS35*, *VPS29*, and *VPS26A*—encode for the three subunits of an endosomal recycling complex known as Retromer (Fig. 2C) (*36*). We were surprised to identify all three Retromer subunits as hits because LENL does not match the consensus recycling motif for Retromer binding, [F/Y/W]x[L/M/V] (fig. S1A) (*37*, *38*), and a [F/Y/W]x[L/M/V] sequence is not present in the cytoplasmic-facing residues of MOR. To confirm the hits from our screen, we identified small interfering RNAs (siRNAs) that caused significant knockdown of individual Retromer subunits or ARF6 in HEK293 cells (fig. S2, A to H). Using these siRNAs, we then measured DOR_MRS(WT)_ down-regulation using the GPCR-APEX2/AUR assay. While we did not detect an effect from ARF6 knockdown, knockdown of any of the three Retromer subunits significantly increased DOR_MRS(WT)_ degradation following 6 hours of agonist treatment (Fig. 2D). Thus, we considered the possibility that the noncanonical LENL recycling motif represents a previously undescribed mechanism for accessing Retromer-dependent trafficking pathways.

### Retromer functions through the bileucine LENL motif to protect MOR from lysosomal down-regulation

Our discovery that all three Retromer subunits oppose OR down-regulation only when the LENL motif is present implicated Retromer as the primary actor at a critical cellular checkpoint of OR regulation. This observation raised several questions: Is Retromer similarly responsible for opposing agonist-induced down-regulation of MOR? If so, where in the cell does Retromer act on MOR, and what is the functional consequence of Retromer activity?

**Fig. 3.**
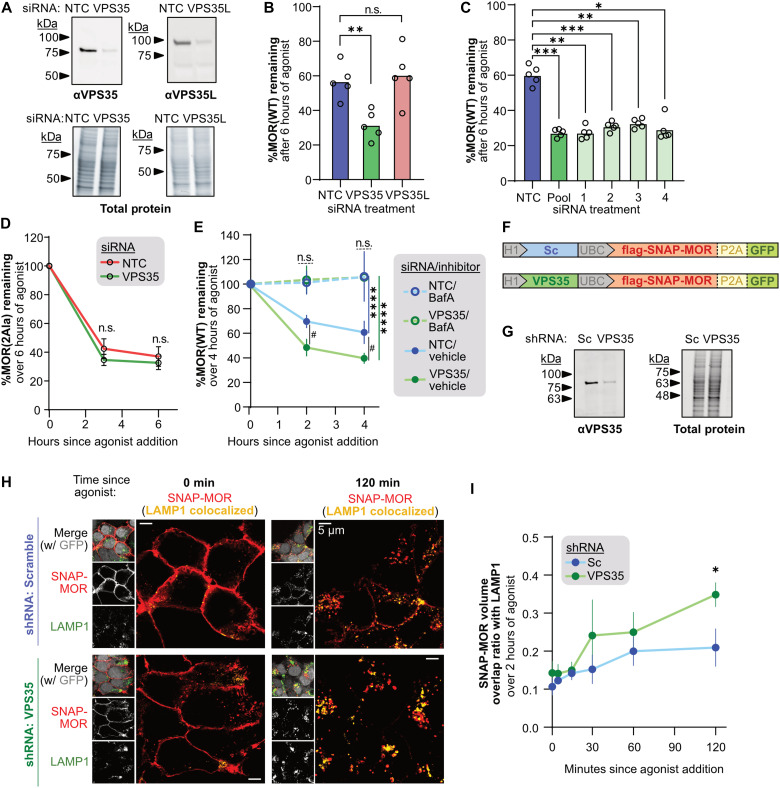
Retromer functions through the bileucine LENL motif to protect MOR from lysosomal down-regulation. (**A**) Representative Western blot for VPS35 and VPS35L following siRNA treatment (*n* = 3). (**B**) Percent MOR(WT) remaining following knockdown of VPS35 or VPS35L and treatment with 10 μM DAMGO (*n* = 5; one-way RM ANOVA with Dunnett’s multiple comparisons: *P* = 0.0061 and 0.7862 for NTC versus VPS35 and VPS35L). (**C**) Same as (B), but after VPS35 knockdown with individual siRNAs (*n* = 5; one-way RM ANOVA with Dunnett’s multiple comparisons: *P* = 0.00020, 0.0023, 0.00070, 0.0026, and 0.011 for NTC versus pool, 1, 2, 3, and 4). (**D**) Percent MOR(2Ala) remaining after VPS35 knockdown and treatment with 10 μM DAMGO (*n* = 5; two-way RM ANOVA with Šidák’s multiple comparisons: *P* = 0.26 and 0.62 for NTC versus VPS35 at 3 and 6 hours, respectively). (**E**) Percent MOR(WT) remaining after VPS35 knockdown and treatment with 3 hours of 100 nM bafilomycin A or vehicle and then 10 μM DAMGO [*n* = 5; three-way RM ANOVA with Tukey’s multiple comparisons: *P* = 0.028 and 0.028 for NTC/Vehicle versus VPS35/vehicle at 2 and 4 hours (#); *P* < 0.0001 for within-siRNA vehicle versus bafilomycin A (BafA) comparisons (****); *P* > 0.9999 for all within-treatment NTC versus VPS35 comparisons (n.s.)]. (**F**) Construct design of Scramble (Sc) or VPS35 shRNA and SNAP-MOR. (**G**) Representative Western blot for VPS35 from cell lysates expressing Sc/SNAP-MOR or VPS35/SNAP-MOR (*n* = 3). (**H**) Representative images of cells expressing Sc or VPS35 shRNA/SNAP-MOR labeled with SNAP-JF549 (red), treated with 10 μM DAMGO, and then fixed and stained for anti-LAMP1 (green). SNAP-MOR colocalized with LAMP1 is shown in yellow (*n* = 3). (**I**) Average overlap ratio of all SNAP-MOR objects with LAMP1 after DAMGO treatment (*n* = 3; two-way RM ANOVA with Šidák’s multiple comparisons: *P* = 0.95, > 0.99, > 0.99, = 0.29, and = 0.039 for Sc versus VPS35 at 0, 5, 15, 30, 60, and 120 min). All experiments are done in stably expressing HEK293-FLP cells. n.s., not significant.

We first asked whether Retromer opposed opioid-dependent down-regulation of MOR. As a pathway-specificity control, we examined the role of a structurally similar but functionally distinct complex, the endosomal recycling complex Retriever. Like Retromer, Retriever is made up of three subunits—VPS29, VPS26C, and VPS35L—but recycles membrane proteins with the recycling motif N[P/x]xY (*39*–*41*). To knockdown function of Retromer and Retriever, we targeted the VPS35 and VPS35L subunits because these subunits are required for assembly of their respective complexes and are not interchangeable (*42*). Pooled siRNAs targeting *VPS35* or *VPS35L* resulted in >80% protein knockdown relative to an NTC (Fig. 3A and fig. S2, A, B, I, and J). We found that knockdown of Retromer function, but not Retriever, resulted in increased opioid-induced MOR down-regulation (Fig. 3B). To verify the specificity of the siRNA pool targeting *VPS35*, we also examined all four individual siRNAs. All four siRNAs efficiently caused VPS35 knockdown (fig. S2, A and B) and led to enhanced opioid-induced down-regulation of MOR (Fig. 3C). Last, to confirm that Retromer was acting in the same pathway as the bileucine recycling motif in MOR, we tested whether combining VPS35 knockdown and MOR_2Ala_ had an additive effect on opioid-induced MOR down-regulation. We found no enhancement in MOR down-regulation when both the LENL motif and Retromer function were disrupted, consistent with a model in which Retromer and the bileucine recycling motif in MOR function in the same pathway (Fig. 3D).

Next, we used two independent methods to determine whether the increase in opioid-induced MOR down-regulation following loss of VPS35 function was due to MOR delivery to lysosomes. First, we used bafilomycin A1, a potent and specific inhibitor of the lysosomal V–adenosine triphosphatase, to disrupt lysosomal function (*43*). We found that pretreatment of cells with bafilomycin A1 completely blocked opioid-induced MOR down-regulation as measured by the GPCR-APEX2/AUR assay in both the presence and absence of VPS35 function, consistent with a model in which agonist-induced MOR down-regulation occurs by delivery of the GPCR to the lysosome (Fig. 3E). Second, to directly visualize opioid-induced delivery of MOR to lysosomes, we developed an imaging strategy to follow MOR trafficking from the cell surface using surface-restricted labeling of soluble *N*-ethylmaleimide–sensitive factor attachment protein (SNAP)–tagged MOR (SNAP-MOR). Specifically, our approach used a single plasmid to deliver to cells both SNAP-MOR and a well-described short hairpin RNA (shRNA) that can knockdown VPS35 expression in human, rat, or mouse cells (Fig. 3F) (*44*, *45*). To validate this approach, we confirmed that the shRNA effectively caused VPS35 knockdown relative to a scrambled shRNA control (Sc) (Fig. 3G and fig. S3, A and B) and that labeling SNAP-tagged MORs with SNAP-Surface-Dye (JF549) does not disrupt their trafficking (fig. S3, C and D). We then tracked the delivery of SNAP-tagged MOR to lysosomes over 120 min of agonist exposure by analyzing the proportion of SNAP-MOR signal that overlapped with endogenous LAMP1, a protein that is highly enriched on lysosomes, in fixed cells using high-resolution Airyscan confocal microscopy. In the absence of agonist treatment, SNAP-MOR was highly localized to the cell surface and did not meaningfully overlap with LAMP1 in either Sc or VPS35 cells (Fig. 3H and fig. S3E). Over the course of agonist treatment, SNAP-MOR overlap with LAMP1-positive structures increased and did so at a faster rate in cells expressing the VPS35 shRNA (Fig. 3, H and I). These results are consistent with VPS35 functioning to oppose delivery of MOR to lysosomes.

Last, we asked whether knockdown of other Retromer subunits would also increase agonist-induced MOR lysosomal down-regulation. Consistent with our observations following loss of VPS35, we saw an increase in MOR_WT_ down-regulation after knockdown of VPS29 and a trending but not significant effect following knockdown of VPS26A (*P* = 0.0549) (fig. S3F). We also examined SNX3, a known Retromer binding protein that aids in recruiting Retromer to endosomes (*46*) and binding cargoes bearing a [F/Y/W]x[L/M/V] motif (*47*), but did not see an effect on MOR lysosomal down-regulation (fig. S3, F to H). Of note, VPS26A and SNX3 have closely related paralogs, VPS26B and SNX12, that may compensate for loss of VPS26A or SNX3 activity (*48*, *49*). Together, these results demonstrate that Retromer and the bileucine motif LENL act together to protect MOR from opioid-induced lysosomal down-regulation.

### The bileucine LENL motif gives MOR access to the Retromer-mediated endosomal recycling pathway

**Fig. 4.**
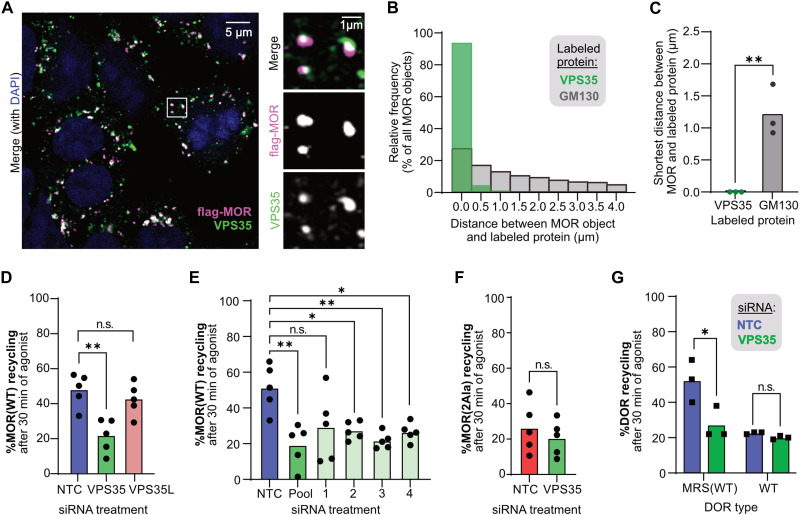
The bileucine LENL motif gives MOR access to the Retromer-mediated endosomal recycling pathway. (**A**) Confocal image of HEK293-FLP cells stably expressing MOR(WT) labeled with anti-FLAG (magenta), treated with 10 μM DAMGO for 20 min, and then fixed and stained for anti-VPS35 (green). Representative image from an *n* = 3 shown. (**B**) Frequency distributions of the closest distance between individual MOR(WT) objects and GM130 or VPS35 objects. (**C**) Average closest distance between MOR objects and a GM130 or VPS35 object (*n* = 3; two-tailed unpaired *t* test: *P* = 0.0061). (**D**) Percent MOR(WT) recycling in stably expressing HEK293-FLP cells treated with siRNAs against VPS35 or VPS35L after 30 min of treatment with 10 μM DAMGO followed by 30 min of 10 μM naloxone, measured by surface receptor staining (*n* = 5; one-way RM ANOVA with Dunnett’s multiple comparisons correction: *P* = 0.0034 and 0.33 for NTC versus VPS35 and VPS35L, respectively). (**E**) Same as (D), but MOR(WT) HEK293-FLP cells were treated with individual or pooled siRNAs against VPS35 (*n* = 5; one-way RM ANOVA with Dunnett’s multiple comparisons correction: *P* = 0.0061, 0.12, 0.015, 0.0081, and 0.034 for NTC versus Pool, 1, 2, 3, and 4, respectively). (**F**) Percent MOR(2Ala) recycling in stably expressing HEK293-FLP cells treated with siRNAs against VPS35 after 30 min of treatment with 10 μM DAMGO followed by 30 min of 10 μM naloxone, measured by surface receptor staining (*n* = 5; two-tailed paired *t* test: *P* = 0.27). (**G**) Percent receptor recycling in stably expressing HEK293-FLP cells treated with siRNAs against VPS35 after 10 μM DADLE followed by 30 min of 10 μM naloxone, measured by surface receptor staining [*n* = 5; two-way RM ANOVA with Šidák’s multiple comparisons correction: *P* = 0.026 for DOR-MRS(WT) NTC versus VPS35; *P* = 0.70 for DOR(WT) NTC versus VPS35]. WT, wild type; n.s., not significant.

We next asked where in the cell Retromer would act on MOR. Given Retromer’s known role in postendocytic sorting, we hypothesized that Retromer would be present on MOR-containing endosomes. Using high-resolution Airyscan confocal microscopy, we observed agonist-dependent colocalization of endogenous Retromer subunit VPS35 and MOR_WT_ in HEK293-FLP cells stably expressing MOR (Fig. 4A and fig. S4A). Specifically, we often saw Retromer adjacent and partially overlapping with MOR, which is consistent with previous observations of GPCR and other membrane cargos that recycle through a SNX27 and/or Retromer-dependent pathway (*13*, *31*, *50*). Pearson’s correlation coefficient analysis supported a higher degree of colocalization between MOR and VPS35 compared with that between MOR and GM130, a Golgi marker that we predicted would not have a meaningful spatial relationship with endocytosed MOR compared with VPS35 over these timescales (fig. S4, B and C) (*51*). To more precisely quantify proximity between MOR and VPS35, we used Imaris image analysis software to render three-dimensional (3D) objects on the basis of MOR (FLAG) and Retromer (VPS35) immunofluorescence from the confocal *z*-stack images. We then calculated the distance between every individual MOR object and its closest Retromer object. We found that most of MOR objects touched a VPS35 object (Fig. 4B). On average, MOR objects were directly in contact with a Retromer object and were 1.50 μm away from a Golgi object (Fig. 4C). Together, these results demonstrate that Retromer is at the correct place and time to mediate MOR postendocytic sorting.

On the basis of these findings, we hypothesized that Retromer acts at endosomes to oppose down-regulation of MOR by specifically sorting MOR into a recycling pathway. To test this hypothesis, we measured the effect of VPS35 knockdown on the ability of MOR to return to the cell surface following agonist-dependent endocytosis and found that loss of Retromer function significantly decreased MOR’s ability to recycle from endosomes (Fig. 4D and fig. S4D). This effect also held true across three out of four individual siRNAs targeting *VPS35* (Fig. 4E and fig. S4E). Consistent with our down-regulation results, knockdown of the functionally distinct complex Retriever had no effect on MOR recycling, demonstrating that the loss of MOR recycling was specific to Retromer (Fig. 4D and fig. S4D). We also examined the effect of the other Retromer subunits and SNX3 and found significant decreases in MOR_WT_ recycling following knockdown of VPS29, VPS26A, or SNX3, although, consistent with our down-regulation assay, the effect size was smaller for the latter two (fig. S4F). These results suggest that Retromer opposes down-regulation of MOR_WT_ by enhancing MOR’s ability to recycle from endosomes after agonist-dependent endocytosis.

Last, we noticed that mutation of LENL or knockdown of Retromer function each blocked about half of MOR recycling over 30 min. As GPCRs can recycle through both recycling-motif–dependent pathways that require recycling complexes and bulk flow pathways that do not, we asked whether Retromer acted in the same pathway as the LENL motif to promote MOR recycling. To test this, we first examined the effect of VPS35 knockdown on the opioid-induced trafficking itinerary of MOR_2Ala_ and saw no additive effect on opioid-induced recycling caused by the mutated recycling motif, suggesting that LENL and Retromer operate in the same pathway (Fig. 4F and fig. S4G). Next, we examined VPS35 knockdown on the recycling of DOR_WT_ and the chimeric DOR_MRS(WT)_ and observed that VPS35 knockdown reduced recycling of the chimeric DOR_MRS(WT)_ to similar levels as DOR_WT_ but had no effect on DOR_WT_ (Fig. 4G). Together, the data demonstrate that Retromer functions through MOR’s bileucine recycling motif to promote MOR recycling from endosomes, thereby slowing the rate of opioid-induced MOR down-regulation at lysosomes.

### Retromer’s role in MOR recycling is conserved in SH-SY5Y cells and rodent striatal neurons

Having shown that Retromer promotes MOR recycling and opposes opioid-induced MOR lysosomal down-regulation in HEK293-FLP cells, we next asked whether its function was conserved across species and cell types. We selected the human neuroblastoma SH-SY5Y line because of its neuronal-like properties and its frequent use as a cellular model for MOR pharmacology (*52*) and primary mouse striatal neurons because the principal spiny projection neurons are known to be important in MOR function and are frequently used as a model system to analyze MOR signaling and trafficking (*44*, *53*, *54*).

**Fig. 5.**
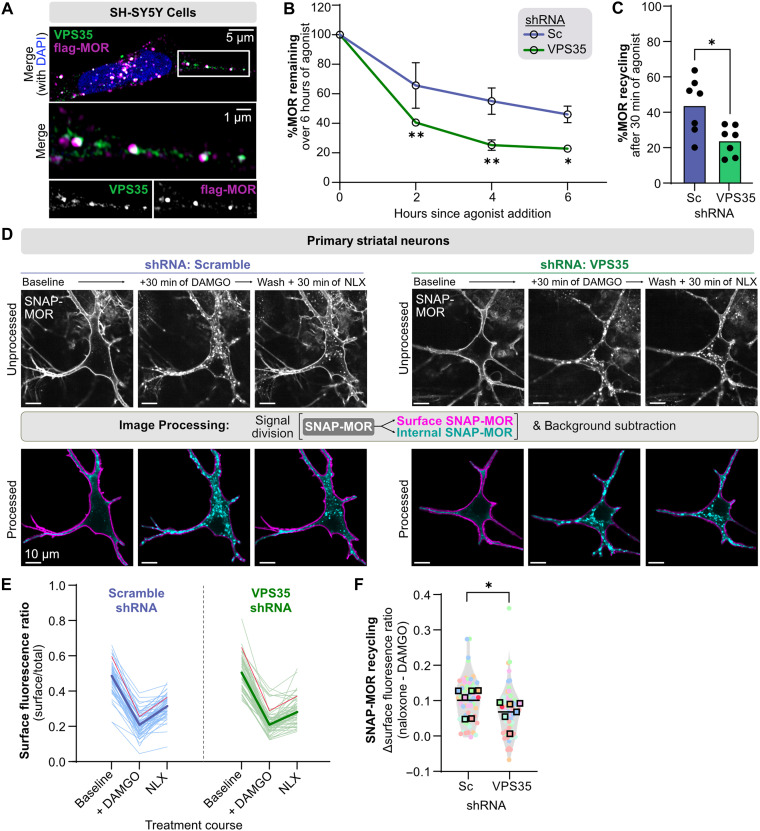
Retromer’s role in MOR recycling is conserved in SH-SY5Y cells and rodent striatal neurons. (**A**) Confocal images of SH-SY5Y cells stably expressing MOR(WT) labeled with anti-FLAG (magenta) and then treated with 10 μM DAMGO for 20 min, fixed, and labeled for anti-VPS35 (green). Representative image shown (*n* = 3). (**B**) Percent MOR(WT) remaining in stably expressing SH-SY5Y cells transduced with Sc or VPS35 shRNA then treated with 10 μM DAMGO for 0, 2, 4, or 6 hours (*n* = 7; two-way RM ANOVA with Šidák’s multiple comparisons correction: *P* = 0.0054, 0.0022, and 0.0081 for Sc versus VPS35 at 2, 4, and 6 hours). (**C**) Percent MOR(WT) recycling in stably expressing SH-SY5Y cells transduced with Sc or VPS35 shRNA and treated with 10 μM DAMGO for 30 min followed by 10 μM naloxone for 30 min (*n* = 7; two-tailed paired *t* test: *P* = 0.021). (**D**) Confocal images of live murine striatal neurons expressing either Sc-shRNA/SNAP-MOR or VPS35-shRNA/SNAP-MOR labeled with 15 min of 1 μM SNAP-JF549 and treated with 30 min of 1 μM DAMGO followed by 30 min of 10 μM naloxone. Top row shows unprocessed images; bottom row shows processed images to separate SNAP-MOR signal into surface (magenta) and internal (cyan). Representative images of *n* = 59 cells (Sc) and *n* = 63 cells (VPS35). (**E**) Quantification of the surface fluorescence ratio of the neurons described in (D). Light lines denote individual neurons. Dark lines denote the medians. Red lines denote the example neurons shown in (D). (**F**) Quantification of SNAP-MOR recycling in neurons as the difference between the surface fluorescence ratio after 30 min of 10 μM naloxone and after 1 μM DAMGO. Circles denote individual neurons color coded by imaging session. Red circles denote example neurons from (D). Squares denote median values for each independent imaging session (*n* = 6; two-tailed paired *t* test: *P* = 0.023).

To monitor MOR trafficking in SH-SY5Y cells, we transduced them to stably express the same flag-MOR_WT_-APEX2 construct used in the HEK293-FLP line (fig. S5A). As in HEK293-FLP cells, we observed VPS35 both adjacent to and partially overlapping with MOR-positive endosomes (Fig. 5A). Both the Pearson’s analysis and the Imaris-based object proximity quantification showed that MOR was consistently found near Retromer following agonist stimulation in SH-SY5Y cells (fig. S5, B to E). To assess the role of Retromer in MOR trafficking, we transduced the MOR_WT_ SH-SY5Y line with the same well-described VPS35 or Scramble shRNA that we used in HEK293 cells (*44*, *45*). We achieved significant VPS35 knockdown 5 days after transduction (fig. S5, F and G). We then asked how knockdown of VPS35 affected MOR trafficking in SH-SY5Y cells and found, as we observed in HEK293-FLP cells, that loss of Retromer function significantly increased the rate at which MOR was down-regulated over 6 hours of agonist treatment and decreased the ability of MOR to recycle (Fig. 5, B and C, and fig. S5H).

Next, we sought to determine whether Retromer was necessary for MOR recycling in mouse primary neurons. For improved health of our primary cultures, we cocultured striatal and cortical cells at a 9:1 ratio and observed abundant expression of *Adora2a* mRNA, a genetic marker of indirect pathway spiny projection neurons (*55*, *56*). *Adora2a* mRNAs were largely absent from cortical cultures, validating that our cocultures were highly enriched for spiny projection neuron principal cells (fig. S6, A and B). We then used our single plasmid delivery approach to simultaneously express VPS35 shRNA (or Scramble control shRNA) and SNAP-MOR and confirmed that the VPS35 shRNA produced significant knockdown of VPS35 and that we could specifically label surface SNAP-MOR using SNAP-Surface-JF549 (fig. S6, C to F). To assess MOR trafficking, we used live-cell confocal microscopy to track the movement of SNAP-MORs within individual neurons over the course of sequential agonist and antagonist treatment. DAMGO treatment induced rapid MOR endocytosis observed as the large shift of SNAP-MOR signal into the cell in both Sc and VPS35 shRNA-expressing neurons (Fig. 5D, middle panels). Subsequently, following antagonist treatment, recycling could be observed in both the depletion of SNAP-MOR–positive endosomes from the cytoplasm and recovery of SNAP-MOR signal on the cell surface (Fig. 5D, right panels). Neurons expressing VPS35 shRNA displayed retained MOR signal at intracellular vesicles and reduced surface signal recovery compared with control neurons (Fig. 5D). To quantify these results, we separated the fluorescent signal from SNAP-MOR on the basis of its localization to either the cell surface or inside the cell. We then divided the surface fluorescence by the total fluorescence (surface fluorescence ratio) to normalize for variations in receptor expression (Fig. 5D, bottom row). Using this approach, we saw that the surface fluorescence ratio decreased following 30 min of agonist treatment and then partially recovered following 30 min of antagonist treatment, representing measurements of MOR internalization and recycling, respectively. This recovery was less pronounced in neurons with reduced VPS35, consistent with a model in which MOR recycling is dependent on Retromer (Fig. 5E). To directly compare receptor recycling between Sc and VPS35 neurons, we determined how much the surface fluorescence ratio increased between the end of agonist treatment (DAMGO) and the end of antagonist treatment (NLX) and found that VPS35 knockdown significantly decreased SNAP-MOR recycling in neurons (Fig. 5F and fig. S6G). These results demonstrate that Retromer’s role in promoting MOR recycling is broadly conserved across a physiologically relevant primary neuron population.

### Clinically relevant opioids display a continuum of dependence on Retromer

Thus far, we focused on a role for Retromer in MOR trafficking following stimulation with the high-efficacy peptide agonist DAMGO, which drives strong MOR endocytosis and entry into the endosomal-lysosomal pathway. However, opioids span a broad range of efficacies. For example, fentanyl and methadone are higher-efficacy opioids and, similar to DAMGO, drive efficient MOR endocytosis (*57*). Other opioids—such as morphine, oxycodone, and buprenorphine—are lower-efficacy MOR agonists. When stimulated with these agonists, MOR undergoes little, or no, endocytosis (*58*, *59*). Given these distinct efficacy-dependent trafficking properties of different opioid ligands, we wanted to interrogate Retromer’s influence on MOR down-regulation following exposure to a panel of different opioids.

**Fig. 6.**
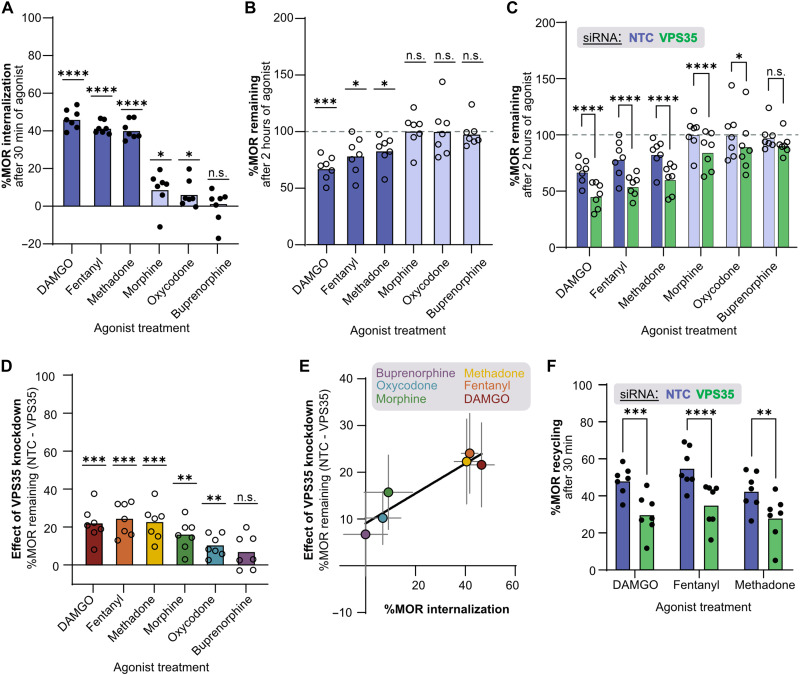
Clinically relevant opioids display a continuum of dependence on Retromer. (**A**) Percent internalization of MOR(WT) in stably expressing HEK293-FLP cells after 30 min of 10 μM agonist treatment measured with surface receptor labeling (*n* = 7; one sample *t* test against 0: *P* < 0.0001 for DAMGO, fentanyl, and methadone; *P* = 0.048 for morphine; *P* = 0.046 for oxycodone; *P* > 0.99 for buprenorphine). Dark blue bars denote high-efficacy agonists and light blue bars denote low-efficacy agonists. (**B**) Percent MOR(WT) remaining in stably expressing HEK293-FLP cells after 2 hours of stimulation with 10 μM agonist measured using the GPCR-APEX2/AUR reaction (*n* = 7; one-sample *t* test against 100: *P* = 0.00020, 0.014, 0.015, 0.85, 0.93, and 0.74 for DAMGO, fentanyl, methadone, morphine, oxycodone, and buprenorphine, respectively). Dashed gray line denotes 100% (i.e., receptor levels before agonist treatment). (**C**) Percent MOR(WT) remaining in stably expressing HEK293-FLP cells after siRNA knockdown of VPS35 and 2 hours of stimulation with 10 μM agonist (*n* = 7, NTC data replotted from Fig. 5B; two-way RM ANOVA with Šidák’s multiple comparisons correction: *P* < 0.0001 for NTC versus VPS35 for DAMGO, fentanyl, methadone, and morphine; *P* = 0.015 and 0.021 for oxycodone and buprenorphine, respectively). (**D**) Effect of VPS35 knockdown on percent MOR remaining for each agonist in stably expressing HEK293-FLP cells (*n* = 7; one sample *t* test against 0: *P* = 0.00070, 0.00030, 0.00060, 0.0020, 0.0031, and 0.093 for DAMGO, fentanyl, methadone, morphine, oxycodone, and buprenorphine, respectively). (**E**) Effect of VPS35 knockdown on percent MOR remaining versus percent MOR internalization induced by each agonist in stably expressing HEK293-FLP cells. (**F**) Percent MOR(WT) recycling in stably expressing HEK293-FLP cells following 30 min of 10 μM agonist treatment and 30 min of 10 μM naloxone treatment measured by surface receptor labeling (*n* = 7; two-way RM ANOVA with Šidák’s multiple comparisons correction; *P* = 0.00020, < 0.0001, and = 0.0021 for DAMGO, fentanyl, and methadone, respectively). n.s., not significant.

Fitting with the literature, stimulation of HEK293-FLP cells stably expressing MOR_WT_ with a saturating dose of fentanyl or methadone induced strong MOR internalization to a similar level as the peptide agonist DAMGO, while stimulation with lower-efficacy opioids induced less internalization (Fig. 6A) (*60*, *61*). Specifically, morphine and oxycodone, but not buprenorphine, induced a small but measurable amount of MOR internalization over 30 min. We next measured how this same panel of opioids drove MOR down-regulation using the GPCR-APEX2/AUR assay. In line with our measurements of MOR internalization, we found that all three higher-efficacy opioids, but not the three lower-efficacy opioids, caused significant MOR down-regulation after 2 hours of agonist exposure (Fig. 6B). Thus, consistent with previous literature, the ability of opioids to induce MOR lysosomal down-regulation is dependent on the ability of these opioids to cause MOR endocytosis.

We next sought to test the hypothesis that Retromer would play an important role in protecting MOR from opioid-induced down-regulation from any opioid that induced substantial MOR endocytosis. As hypothesized, we found that Retromer knockdown resulted in an increase in MOR down-regulation following exposure to the high-efficacy opioids fentanyl and methadone (Fig. 6C). We also observed a small but significant increase in MOR down-regulation in cells lacking Retromer with the two low-efficacy opioids—morphine and oxycodone—which could induce measurable MOR internalization (Fig. 6C). This result was notable as we had been unable to measure significant MOR down-regulation in response to these opioids in wild-type conditions, suggesting that VPS35 knockdown revealed a small but significant dependency on Retromer that had been likely masked by a combination of low levels of endocytosis and new MOR synthesis.

To look at this effect more directly, we reanalyzed the down-regulation experiment to isolate the effect of Retromer knockdown on MOR down-regulation and found that all opioids tested, with the exception of buprenorphine, which does not drive detectable MOR endocytosis, showed significantly greater down-regulation following Retromer knockdown (Fig. 6D). There was a linear relationship between the amount of endocytosis driven by each opioid and the dependence of MOR on Retromer to avoid down-regulation (Fig. 6E). Thus, the relative effect of Retromer knockdown on MOR lysosomal down-regulation was directly proportional to the amount of internalization induced by a given agonist, consistent with our results showing that Retromer regulates MOR after endocytosis. Last, we asked whether VPS35 knockdown reduced MOR recycling for the higher-efficacy opioids that drove sufficient MOR endocytosis to allow for a reliable measurement of recycling. We found that like DAMGO, MOR stimulated by either fentanyl or methadone was dependent on Retromer for recycling (Fig. 6F). Together, these results demonstrate that Retromer protects MOR from lysosomal down-regulation following exposure to multiple opioids, including both higher- and lower-efficacy opioids, to a degree dependent on opioid efficacy and MOR endocytosis.

### Bileucine recycling motifs are a general mechanism for accessing Retromer-dependent recycling

Our results in this study have defined a role for Retromer in protecting MOR from opioid-induced down-regulation by promoting MOR recycling from endosomes. Additionally, our study demonstrates that the bileucine LENL recycling motif in MOR, which was previously considered to be a noncanonical recycling motif due to its lack of sequence conservation with other recycling motifs (*5*, *23*), is a mechanism to gain access to the Retromer recycling pathway. These results raised a question: Are bileucine (LxxL) motifs for Retromer-based recycling unique to MOR or is this mechanism also used by other membrane proteins?

**Fig. 7.**
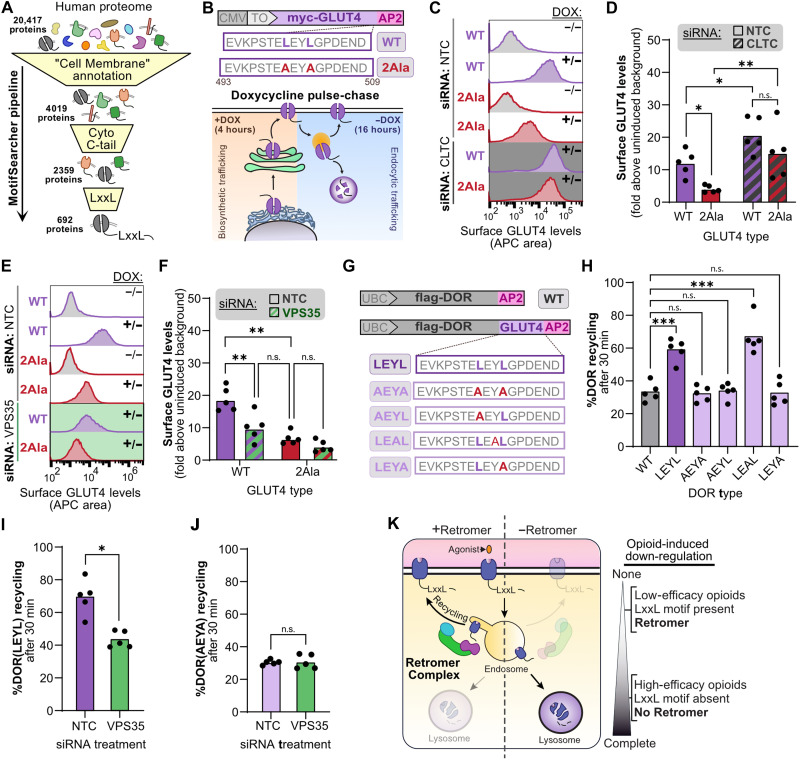
Bileucine recycling motifs are a general mechanism for accessing Retromer-dependent recycling. (**A**) Cartoon depicting workflow for identifying proteins with LxxL motifs. (**B**) Construct design of GLUT4(WT) and GLUT4(2Ala) under the CMV-TetO2 promoter and cartoon of doxycycline pulse-chase protocol. (**C**) Representative histograms of GLUT4 surface expression in HEK293-TREx cells, with or without doxycycline and siRNA knockdown of clathrin (CLTC) (*n* = 5). (**D**) Quantification of surface GLUT4 levels following siRNA knockdown of CLTC (*n* = 5; two-way RM ANOVA with Fisher’s least significant difference (LSD) test: *P* = 0.024 for WT NTC versus 2Ala NTC; *P* = 0.019 for WT NTC versus WT CLTC; *P* = 0.0081 for 2Ala NTC versus 2Ala CLTC; *P* = 0.071 for WT CLTC versus 2Ala CLTC). (**E**) Representative histograms of GLUT4 surface expression in HEK293-TREx cells, with or without doxycycline and siRNA knockdown of VPS35 (*n* = 5). (**F**) Quantification of surface GLUT4 levels following siRNA knockdown of VPS35 (*n* = 5; two-way RM ANOVA with Fisher’s LSD test: *P* = 0.0015 for WT NTC versus 2Ala NTC; *P* = 0.0052 for WT NTC versus WT VPS35; *P* = 0.19 for 2Ala NTC versus 2Ala VPS35; *P* = 0.10 for WT VPS35 versus 2Ala NTC). (**G**) Construct design of DOR chimeras with appended GLUT4 tails, with or without leucine mutations. (**H**) Percent DOR recycling in stably expressing HEK293-FLP cells after 10 μM DADLE treatment and 30 min of 10 μM naloxone treatment [*n* = 5; one-way RM ANOVA Dunnett’s multiple comparisons correction: *P* = 0.00030, = 0.93, = 0.99, = 0.00070, and >0.99 for DOR(WT) versus DOR(LEYL), DOR(AEYA), DOR(AEYL), DOR(LEAL), and DOR(LEYA), respectively]. (**I**) Percent DOR(LEYL) recycling following siRNA knockdown of VPS35 and treatment with 10 μM DADLE for 30 min followed by 10 μM naloxone for 30 min measured (*n* = 5; two-tailed paired *t* test: *P* = 0.013). (**J**) Same as (I), but in HEK293-FLP cells expressing DOR(AEYA) (*n* = 5; two-tailed paired *t* test: *P* = 0.86). (**K**) Working model describing how Retromer acts through the bileucine LxxL recycling motif. WT, wild type; n.s., not significant.

To identify proteins that potentially traffic through a similar bileucine/Retromer pathway, we developed a custom application, MotifSearcher, that parses through a user-provided list of UniProt Knowledgebase protein identifiers and searches for a specified amino acid sequence within designated topological stretches of a protein. We used the UniProt Knowledgebase to create a list of all verified “cell membrane” human proteins that have a cytoplasmic C terminus. We then used MotifSearcher to search for LxxL motifs found only within cytoplasmic-facing regions of the last 100 amino acids of these 2359 proteins (Fig. 7A).

There were 692 unique proteins with a bileucine LxxL sequence in their cytoplasmic-facing regions, most of which were signaling receptors and transporters (fig. S7A and table S3). As a point of comparison, we used MotifSearcher to perform the same analysis for other previously described recycling motifs which function with Retromer, ESCPE-1, or Retriever: [F/Y/W]x[L/M/V], [D/E][S/T]xΦ-COOH, Φx[F/Y/V]x[F/Y], and N[P/x]xY (table S3) (*22*, *62*, *63*). These motifs were present in the cytoplasmic-facing regions of 986, 124, 436, and 155 unique proteins, respectively, and were found mostly in signaling receptors and transporters (fig. S7, B and C).

We next asked whether Retromer-dependent trafficking had been described for any of the identified LxxL-containing proteins. As previous research has suggested that Retromer-dependent recycling motifs need to be a minimum distance from the membrane to successfully engage Retromer (≥15 amino acids) (*38*), we further parsed our list of LxxL-containing proteins into 430 proteins that met this criterion. Notably, we identified several proteins from this list that have already been linked to Retromer in an unbiased screen for proteins that depend on VPS35 for their stable surface expression (e.g., adenylyl cyclase 9: 1344-LTKL; SLC12A7: 1058-LEVL; and PLXNA1: 1867-LAAL) (*64*). We also identified an LxxL motif in the C-terminal tail of the glucose transporter 4 (GLUT4: 500-LEYL), which undergoes complex intracellular trafficking in response to insulin availability (*65*, *66*). Several independent studies identified that a portion of the GLUT4 C-terminal tail containing the LxxL sequence (498-TELEYLGP) is important in its postendocytic trafficking, with both the glutamate and leucine residues showing effects upon mutation (*67*–*70*). However, the precise function of this motif in the GLUT4 postendocytic pathway was unclear. Because GLUT4 postendocytic sorting has also been shown to require Retromer (*71*, *72*), we hypothesized that the bileucine sequence in the GLUT4 C-terminal tail is a bona fide recycling motif that is both necessary and sufficient to provide entry into the Retromer recycling pathway.

To first test the necessity of the bileucine motif and its connection to Retromer in GLUT4 postendocytic trafficking, we needed to adjust our approach because GLUT4, unlike MOR and many other GPCRs, undergoes constitutive endocytosis from the plasma membrane rather than agonist-dependent endocytosis (*73*). Because the classical function of recycling motifs is to stabilize surface expression of membrane proteins in a manner dependent on endocytosis, we developed a pulse-chase style experiment where we could monitor the levels of GLUT4 on the cell surface under conditions where we genetically blocked different steps in the GLUT4 trafficking pathway (e.g., endocytosis and recycling) (Fig. 7B). Specifically, we created constructs for doxycycline-inducible expression of either wild-type GLUT4 or a mutant GLUT4 in which the two leucines of the LEYL sequence had been mutated to alanines [myc-GLUT4_(WT/2Ala)_-APEX2]. Both constructs also contained an exofacial myc tag to quantify GLUT4 cell surface expression (Fig. 7B). To be able to directly compare the surface expression between these constructs, we used engineered cells (HEK293 T-REx) that contain both a tetracycline repressor protein and a single flippase recognition target (FRT) site in the genome to integrate the construct of interest.

Consistent with our hypothesis that the bileucine sequence in GLUT4 can function as a recycling motif, we found that the steady state surface expression of GLUT4_2Ala_ was approximately threefold lower than that of GLUT4_WT_ (Fig. 7, C and D). To determine whether the function of the bileucine motif in GLUT4 was postendocytic, we also conducted these measurements in cells in which GLUT4 endocytosis was blunted following siRNA-mediated knockdown of clathrin (CLTC; fig. S7, D and E) (*74*–*76*). Under these conditions, GLUT4 surface expression increased for both GLUT4_WT_ and GLUT4_2Ala_, and the difference between the wild-type and the bileucine mutant largely erased, suggesting that activity of the LEYL bileucine sequence on GLUT4 trafficking is postendocytic (Fig. 7, C and D). We next asked whether Retromer functioned through the bileucine motif to promote GLUT4 surface expression. We found that knockdown of VPS35 resulted in a large reduction in GLUT4_WT_ expression on the surface and that there was no significant additive effect on GLUT4_2Ala_ surface expression in cells with VPS35 knocked down, consistent with a model in which LEYL sequence in GLUT4 and Retromer function in the same pathway (Fig. 7, E and F). Together, these results demonstrate that the bileucine sequence in the GLUT4 C terminus functions with Retromer to maintain surface expression of the transporter in an analogous mechanism to the MOR bileucine sequence.

To next test the sufficiency of the GLUT4 bileucine motif to promote Retromer-dependent recycling, we created chimeric constructs in which we fused the final 17 amino acids of GLUT4 to DOR in a manner analogous to our previous DOR_MRS_ constructs [flag-DOR_GLUT4(LEYL)_-APEX2]. We also created DOR_GLUT4_ chimeras in which either one or both leucines were mutated to alanines [flag-DOR_GLUT4(AEYA/AEYL/LEYA)_-APEX2]. Additionally, as a negative control, we examined the effects of mutating the tyrosine residue to an alanine, as this residue had not been directly implicated in the postendocytic sorting of GLUT4 [flag-DOR_GLUT4(LEAL)_-APEX2] (Fig. 7G) (*68*, *70*, *77*). The DOR_GLUT4(LEYL)_ chimera showed no defects in surface expression, signaling, or agonist-induced internalization (fig. S7, F to H). We then examined recycling and found that the fragment of the GLUT4 C-terminal tail containing its bileucine sequence could induce recycling of DOR (Fig. 7H) only if, like the LENL sequence in MOR, both leucines in the LEYL sequence were intact. Additionally, we observed no effect from mutating the tyrosine, suggesting that the LEYL sequence in GLUT4 can act as a bona fide LxxL recycling motif (Fig. 7H). We then assessed whether the GLUT4 LEYL motif induced Retromer-dependent trafficking by knocking down VPS35 in cells expressing DOR_GLUT4(LEYL)_. We found that knockdown of Retromer decreased the ability of the GLUT4 tail to induce recycling (Fig. 7I and fig. S7I). Knockdown of VPS35 had no effect on recycling of DOR_GLUT(AEYA)_ that lacks a functional bileucine motif, consistent with a model in which the bileucine motif from GLUT4 and Retromer function in the same pathway (Fig. 7J and fig. S7J). Together, these results demonstrate that the bileucine motif in GLUT4 is a necessary and sufficient recycling motif that allows entry of GLUT4 into the Retromer pathway. Broadly, our findings suggest that LxxL motifs represent a distinct type of Retromer recycling motif and allow a larger portion of the membrane proteome to access Retromer-dependent trafficking pathways than previously appreciated. This pathway plays a critical role in protecting ORs from lysosomal down-regulation following prolonged exposure to high-efficacy opioids (Fig. 7K).

## DISCUSSION

Agonist-induced trafficking plays a critical role in the regulation of many GPCRs. Sequence-dependent GPCR recycling from endosomes protects the receptor from rapid agonist-induced lysosomal down-regulation (*8*, *13*, *21*, *78*, *79*). However, many GPCRs that can undergo sequence-dependent recycling lack canonical recycling motifs, and, thus, the mechanisms of their trafficking remain unclear (*5*). One such GPCR is the MOR, which has a noncanonical bileucine recycling motif (LENL) and, consequently, an unresolved mechanism for how the cell regulates this receptor following endocytosis (*21*). Addressing this gap is of potential therapeutic relevance as high-efficacy opioids like fentanyl drive MOR lysosomal down-regulation, and this process has been linked to the development of pharmacological opioid tolerance, a phenomenon that limits the utility of opioids in chronic pain treatment (*61*, *80*) and contributes to opioid toxicity (*81*–*84*).

### Cellular mechanisms regulating MOR function in response to high-efficacy opioids and implications for opioid drug tolerance

A fundamental biological question is how cells adapt to chronic opioid exposure. One type of adaptive mechanism occurs at the level of the MOR where the exposure to strong opioids like fentanyl or methadone causes GPCR kinase (GRK) 2/3-mediated hierarchical phosphorylation of MOR, efficient binding of β-arrestin to MOR, and MOR endocytosis (*60*). Our study reveals the next step of this process: At endosomes, MOR is recycled back to the plasma membrane by the Retromer complex. The ability of MOR to access the Retromer recycling pathway is completely dependent on its bileucine recycling sequence LENL, which, before our study, was considered a noncanonical motif because of its lack of similarity to other known recycling motifs (*5*, *21*, *23*). Consequently, Retromer stabilizes MOR in cells in the presence of chronic opioid exposure by removing the receptor from the endosomal-lysosomal pathway and returning it to the cell surface. It is interesting to note that, even in the presence of a functional LENL/Retromer pathway, MOR still undergoes down-regulation, although at a much slower rate. Thus, it is likely that other pathways that promote MOR down-regulation, including the previously identified ubiquitin-dependent ESCRT complexes, may compete with Retromer for the fate of MOR at endosomes (*85*).

In the context of a larger genetic picture of MOR postendocytic sorting, it is notable that our CRISPRi screen identified other genes that are likely to be involved in trafficking of MOR including those likely to function in the endosomal-lysosomal pathway (*WDR91* and *RPTOR*), at the Golgi (*RHOBTB3* and *TRAPPC11*), and from the plasma membrane (*ARF6* and *GAPVD1*). Of these genes, *ARF6* has been previously linked to MOR trafficking, with known roles in regulating GPCR endocytosis and recycling (*86*, *87*). Consistent with our previous study, we found that CRISPR-based genetic screens do not identify every gene involved in regulatory pathways due to genetic compensation or insufficient sgRNA activity (*24*, *88*). For example, we did not identify GRKs, β-arrestin, or dynamin as hits, although these have been previously linked to MOR trafficking (*60*). Last, we found that, while our comparative-based screen approach effectively identified trafficking genes specific to LENL including Retromer, *ARF6*, *RAPTOR*, and *TRAPC11*, it also identified genes known to control transcription, which we had initially anticipated would be shared between the two screens. While we did not investigate these transcriptional genes, we anticipate that these genes are likely general regulators of GPCR expression that were identified in one screen, rather than both, because of differences in screen performance (e.g., relative efficacy of the CRISPRi knockdown between the independent cell lines) or design (e.g., seven sublibraries versus three sublibraries and 500-fold versus 300-fold sgRNA coverage).

One of the intriguing features of opioids is that the cellular mechanisms that regulate MOR differ when MOR is stimulated by high-efficacy opioid agonists compared with lower-efficacy opioid agonists (*59*, *61*). Lower-efficacy opioids cause minimal GRK-based phosphorylation of MOR, poor binding between MOR and β-arrestin, and little MOR endocytosis (*60*, *89*, *90*). Instead, other kinases including GRK5, protein kinase C, and c-Jun N-terminal kinase regulate MOR when stimulated by lower-efficacy opioids, presumably in a trafficking-independent manner (*60*). Our work is largely consistent with this model. We found that lower-efficacy opioids cause small (morphine and oxycodone) or no (buprenorphine) MOR internalization over short-term timescales and no measurable down-regulation over longer timescales likely because new receptor synthesis outpaces any down-regulation. Retromer knockdown revealed that a small population of MORs do undergo lysosomal down-regulation following stimulation with morphine or oxycodone, suggesting that Retromer likely plays a role in protecting MOR from down-regulation from any opioid that can drive MOR endocytosis. In this light, it is interesting to note that, while morphine drives minimal MOR endocytosis in most cultured cell models, morphine can drive greater MOR endocytosis in some types of neuronal populations, thus raising the potential for a tissue-specific dependence on MOR for Retromer function (*91*). An additional variable to consider for OR trafficking in vivo is the potential role of MOR splice variants. On the RNA level, there is evidence for multiple splice variants of MOR including several variants that specifically replace the last exon, which contains the bileucine LENL motif, with sequences that do not promote MOR recycling (*92*–*94*). Thus, it is intriguing to consider the potential that nature has evolved mechanisms to stabilize or destabilize agonist-stimulated MOR through RNA splicing to add or remove a functional connection between MOR and Retromer. However, it is important to note that a recent study using a knock-in mouse model did not find evidence for expression of these MOR splice variants on a protein level in whole-brain extract from opioid naïve animals (*95*). Together, our data support the consensus model that two very different cellular programs are responsible for MOR regulation when activated by opioids—a trafficking-based program using GRKs, β-arrestin, and Retromer for high-efficacy agonists and a distinct program—that is largely but not completely independent of postendocytic trafficking—for lower-efficacy agonists.

A long-standing question in the opioid field is what role these trafficking processes play in the development of opioid tolerance (*96*). This question is particularly interesting because, while only high-efficacy opioids cause measurable MOR down-regulation in vivo (*3*, *97*–*99*), both low- and high-efficacy opioids can drive pharmacological tolerance (*20*, *100*–*102*). While this question is far from resolved, several lines of evidence support a model that MOR postendocytic trafficking affects the development of opioid tolerance in vivo in contexts where MOR endocytosis can occur. First, mice expressing mutant MORs, which lack a large part of the receptor C-terminus including the LENL motif, develop tolerance to high-efficacy opioids faster than wild-type mice (*20*). Second, mice expressing mutant MORs that gain the ability to undergo substantial endocytosis in response to morphine develop tolerance more slowly when the LENL motif is present in MOR compared with when it is absent (*19*, *20*). These genetic studies suggest that MOR endocytosis can promote tolerance but that this is opposed by MOR recycling (*103*). Here, we define Retromer as the first molecular target for motif-dependent MOR recycling and thus pave the way for future studies to determine whether Retromer plays a role in tolerance development.

### Diversity of endosomal recycling pathways and implications for Retromer function

Classically, membrane protein recycling was thought to occur through a sequence-independent bulk flow pathway (*33*). However, it is now clear that many membrane proteins have cis-acting recycling motifs, often in their C-terminal tails, that promote their recycling by binding to endosomal recycling complexes and sorting into endosomal tubules (*31*, *104*). One example is the Retromer complex (VPS35/VPS29/VPS26A), which binds to the consensus sorting motif [F/Y/W]x[L/M/V] (*22*, *37*, *38*, *104*, *105*). In the past 15 years, multiple previously unknown adaptors, cargo binding complexes, and recycling motifs have been identified in mammalian cells. These include SNX27/Retromer ([D/E][S/T]xΦ-COOH) (*10*, *13*, *63*, *64*), SNX17/Retriever (N[P/x]x[F/Y]) (*39*, *106*, *107*), and SNX-BAR/ESCPE-1 (Φx[F/Y/V]x[F/Y]) (*108*, *109*). However, even this expanded understanding of the diversity of recycling motifs and complexes cannot explain the recycling of many GPCRs and other membrane proteins (*5*).

Here, we demonstrate that bileucine recycling motifs are a distinct mechanism by which membrane proteins can access the Retromer recycling pathway, including MOR and GLUT4, and identified additional candidate proteins with a custom bioinformatics pipeline MotifSearcher. While we do not anticipate that all the identified proteins recycle in a manner dependent on the identified motif and Retromer, we note that several of the proteins that have C-terminal bileucine LxxL sequences were already linked to Retromer function (*64*, *71*, *72*). We focused on GLUT4 because its postendocytic sorting had been shown by independent groups to be dependent on both Retromer and its C-terminal tail (*67*, *70*–*72*). Although no consensus Retromer binding motif had been identified in GLUT4, mutational analysis had shown that amino acids in the sequence TELEYLGP in its C-terminal tail affected its postendocytic trafficking (*68*, *69*, *70*, *77*).

We show here that the bileucine motif in the GLUT4 C-terminal tail is both necessary and sufficient to allow access into the Retromer-dependent trafficking pathway. It is important to note that our model cell line, an engineered version of HEK293 cells, lacks the insulin-dependent surface expression that defines a critical step of GLUT4 trafficking, and, thus, future studies will be required to determine how the connection between Retromer and the GLUT4 bileucine motif that we define here shapes GLUT4 trafficking in insulin-responsive cells. Additionally, it is likely that other determinants outside the LxxL/Retromer genetic connection are important in GLUT4 trafficking. For example, one study demonstrated that the VPS10-like protein sortillin can act as an adaptor on the luminal side of endosomes to stabilize a connection between GLUT4 and Retromer on the cytoplasmic side (*71*). Additionally, while the recycling sequences in both MOR and GLUT4 contain glutamates (MOR: QLENLE; and GLUT4: ELEYLG), the contribution of these acidic residues to recycling motif function is not identical: Previous work has shown that these glutamate residues contribute to postendocytic trafficking of GLUT4 but are dispensable for recycling of MOR (*21*, *67*, *68*). Thus, while our study demonstrates that the LxxL sequence represents a core recycling motif that gives access to Retromer-dependent trafficking, it is likely that additional endosomal proteins and receptor features also contribute to recycling.

An open question is how bileucine-based recycling motifs allow access to the endosomal Retromer recycling pathway. The consensus model for Retromer function is that VPS26 acts as a membrane proximal cargo-binding subunit, which is linked to the membrane distal regulatory subunit VPS29 by a VPS35 scaffold (*110*–*112*). The interaction between Retromer and membrane proteins containing a Retromer-based recycling motif serves to enrich membrane proteins into endosomal recycling tubules, which undergo membrane scission to form transport vesicles for recycling to either the Golgi or plasma membrane (*31*, *50*). A recent structural study has provided further insight into how VPS26 binds cargo containing the consensus motif [F/Y/W]x[L/M/V] (*47*). This structure resolved an extensive set of contacts between VPS26, the recycling motif from DMT1-II (551-QPEL**Y**L**L**-557), and Retromer binding protein SNX3. These contacts were centered around a critical interaction of L557 in DMT1-II with a hydrophobic pocket in VPS26. Notably, this structure revealed that residues in the DMT1-II tail outside the consensus motif (YLL) are important in binding SNX3/Retromer including L554 that also engages VPS26. Intriguingly, we noted that L554 and L557 in DMT1-II form a bileucine LxxL sequence, and cell biological studies of DMT1-II recycling have shown that both leucines are able to contribute to DMT1-II trafficking, with L557 playing a more critical role (*38*). Thus, it is possible that MOR and DMT1-II use structurally and functionally overlapping, but not identical, mechanisms for recycling. While future studies will be required to determine whether MOR or other membrane proteins with LxxL motifs directly bind Retromer, our previous work using time-resolved proximity labeling captured Retromer subunits in the MOR proximal proteome following stimulation with high-efficacy agonists, suggesting that Retromer is specifically enriched near MOR on endosomes (*26*). Together, our findings here have identified a distinct type of endosomal recycling motif that provides access the endosomal Retromer recycling pathway and raises the potential that a broader portion of the membrane proteome uses the Retromer pathway than previously appreciated.

## MATERIALS AND METHODS

### Chemicals

DAMGO acetate salt (E7384) and DADLE acetate salt (E7131) were purchased from Sigma-Aldrich. Naloxone hydrochloride (0599) was purchased from Tocris. Fentanyl, methadone, morphine, oxycodone, and buprenorphine were obtained through the National Institute on Drug Abuse Drug Supply Program. All drugs were resuspended at 10 mM in double-distilled water and stored as frozen aliquots at −20°C. AUR (A36006) was purchased from Thermo Fisher Scientific, resuspended at 10 mM in anhydrous dimethyl sulfoxide (DMSO), and stored as frozen aliquots at −20°C. Hydrogen peroxide [30% (w/w)] (H1009) was purchased from Sigma-Aldrich, stored at 4°C, and diluted in double-distilled water immediately before use. Sodium ascorbate (A7631) was purchased from Sigma-Aldrich, stored at 4°C, and resuspended at 1 M in double-distilled water immediately before use. Bovine serum albumin (BSA), fatty acid free, (A7030) was purchased from Sigma-Aldrich and resuspended in phosphate-buffered saline (PBS). Bafilomycin A (B1793) was purchased from Sigma-Aldrich, resuspended at 100 μM in anhydrous DMSO, and stored as frozen aliquots at −20°C. SNAP-Surface Block (S9143S) was purchased from New England Biolabs (NEB), resuspended at 1 mM in anhydrous DMSO, and stored as frozen aliquots at −20°C. SNAP-JF549 was a gift from L. Lavis (Janelia) and was handled as described for SNAP-Surface Block. Doxycycline hyclate (AAJ6057914) was purchased from Thermo Fisher Scientific, resuspended at 1 mg/ml in double-distilled water, and stored at −20°C. Thawed aliquots in active use were kept at 4°C for no longer than 1 month.

### Antibodies

M1 anti-FLAG (Sigma-Aldrich, catalog no. F3040; RRID: AB_439712) was used at 1:500 to 1:1000 for immunofluorescence. M1 was conjugated to Alexa Fluor 647 (M1-647) using an amine-reactive labeling kit (A20173) from Thermo Fisher Scientific and used at 1:1000 to 1:2000 for flow cytometry. Anti-VPS35 (Novus, catalog no. NB 100-1397; RRID: AB_527526) was purchased at 1:500 for immunofluorescence and 1:1000 for HEK293 and neuron Western blots. Anti-VPS29 (ab236796) was purchased from Abcam and used at 1:1000. Anti-VPS26A (Abcam, catalog no. ab23892; RRID: AB_2215043) was used at 1:1000. Anti-Arf6 (Cell Signaling Technology, catalog no. 5740; RRID: AB_10694539) was used at 1:500. Anti-VPS35L (Abcam, catalog no. ab97889; RRID: AB_10679218) was used at 1:1000. Anti-LAMP1 (Cell Signaling Technology, catalog no. 15665; RRID: AB_2798750) was used at 1:200 for immunofluorescence. Anti-SNX3 (ab314492) was purchased from Abcam and used at 1:1000. Anti-VPS35 (Abcam, catalog no. ab157220; RRID: AB_2636885) was used at 1:1000 for SH-SY5Y Western blots. Anti-myc (Cell Signaling Technology, catalog no. 2276; RRID: AB_331783) was used at 1:1000 for flow cytometry. Anti-CLTC (Santa Cruz Biotechnology, catalog no. sc-12734; RRID: AB_627263) was used at 1:1000 for Western blots. Anti-GM130 (12480S) was purchased from Cell Signaling Technology and used at 1:500 for immunofluorescence. Donkey anti-mouse 647 (A31571), donkey anti-goat 488 (A11055), and donkey anti-rabbit 488 (A21206) were purchased from Invitrogen and used at 1:1000 for immunofluorescence. Donkey anti-goat 647 (A32849) was purchased from Invitrogen and used at 1:1000 for immunofluorescence and 1:2500 for Western blots. StarBright Blue 700 goat anti-rabbit (12004161) and goat anti-mouse (12004158) were purchased from Bio-Rad and used at 1:2500 for Western blots.

### Complementary DNA constructs

UBC:MOR_WT_-APEX2 is encoded by the construct puDNA5-SSF(signal sequence FLAG)–MOR_WT_–APEX2, which was created by cutting puDNA5 at the Nhe I and Bam HI restriction sites and inserting MOR_WT_, which was amplified by polymerase chain reaction (PCR) from pSYN-MOR_WT_, and a linker (GGGSGGG) with APEX2, which was encoded by a gBlock. UBC:MOR_2Ala_-APEX2 was created by cutting puDNA5 at the Nhe I and Bam HI restriction sites and inserting MOR2ala and linker-APEX2, which were both amplified by PCR from the UBC:MOR_WT_-APEX2 construct. During PCR amplification, L407 and L410 in the MOR_WT_ sequence were replaced with alanines. UBC:DOR_WT_-APEX2 is encoded by puDNA5-SSF-DOR-APEX2. UBC:DOR_MRS(WT)_-APEX2 was created by cutting puDNA5 at the Nhe I and Bam HI restriction sites and inserting the DOR_WT_ sequence, which was PCR amplified from pSyn-DOR_WT_-APEX2 and a sequence containing the last 17 amino acids of MOR_WT_, a linker, and the APEX2 tag, which was encoded by a gBlock. UBC:DOR_MRS(2Ala)_ was created by cutting puDNA5-DOR_WT_ with Bam HI and inserting the sequence for the last 17 amino acids of MOR_WT_ with the two leucines mutated to alanines and a linker, which was encoded by a gBlock, and the sequence for APEX2, which was amplified by PCR from UBC:DOR_MRS(WT)_. pLenti UBC:MOR_WT_-APEX2 was created by cutting pSYN-MOR-APEX2 at the Pac I and Xba I restriction sites to remove the SYN promoter and inserting the UBC promoter, which was PCR amplified from pUBC-MOR-APEX2-Puro. GloSensor20F-IRES-Rluc was previously described (*113*). pLenti Scramble shRNA and pLenti VPS35 shRNA were gifts from P. Temkin (Biogen) and M. von Zastrow (University of California, San Francisco). H1:Sc-shRNA/UBC:SSF-SNAP-MOR_WT_ and H1:VPS35 shRNA/UBC:SSF-SNAP-MOR_WT_ were created by cutting pLenti Scramble or VPS35 shRNA with Bam HI and Xho I and inserting SSF-SNAP-MOR_WT_, which was PCR-ed from a commercially produced AAV vector encoding the insert (Virovek). CMV-TO:myc-GLUT4_WT_-APEX2 was created by cutting pCDNA5-TO-FRT with a custom multiple cloning site inserted by PCR with Nhe I and Xho I and inserting myc-GLUT4_WT_ and APEX2 by PCR amplifying from pLenti-myc-GLUT4-mCherry and UBC:MOR_WT_-APEX2, respectively. CMV-TO:myc-GLUT4_2Ala_ was created in the same way, with alanine mutations inserted during PCR amplification. pLenti-myc-GLUT4-mCherry was a gift from W. Han (Addgene, plasmid no. 64049) (*114*). UBC:DOR_GLUT4(LEYL)_-APEX2 was created by cutting puDNA5 at the Nhe I and Xho I restriction sites and inserting DOR, which was PCR amplified from UBC:DOR_WT_-APEX2, the final 17 amino acids from GLUT4, which was PCR amplified from pLenti-myc-GLUT4-mCherry, and APEX2, which was PCR amplified from UBC:DOR_WT_-APEX2. UBC:DOR_GLUT4(AEYA/AEYL/LEAL/LEYA)_-APEX2 were created by cutting puDNA5 at the Nhe I and Xho I restriction sites and inserting DOR_GLUT4_ with the corresponding alanine mutations and APEX2, which were both PCR amplified from UBC:DOR_GLUT4(LEYL)_-APEX2. All cDNAs code for the mouse protein (MOR: UniProt, accession code P42866; DOR: UniProt, accession code P32300; and GLUT4: UniProt, accession code P14142).

### Cell culture and stable cell line generation

FLP-In-293 (HEK293-FLP, R75007) cells and FLP-In-TREx 293 cells (HEK293 TREx, R78007) were purchased from Thermo Fisher Scientific and grown in Dulbecco’s modified Eagle’s medium (DMEM; Thermo Fisher Scientific, 11965-092) supplemented with 10% fetal bovine serum (FBS; Cytiva) at 37°C and 5% CO_2_. Stable cell lines were created by transiently transfecting either UBC:MOR_WT_-APEX2, UBC:MOR_2Ala_-APEX2, UBC:DOR_WT_-APEX2, UBC:DOR_MRS(WT)_-APEX2, UBC:DOR_MRS(2Ala)_-APEX2, CMV-TO:GLUT4_WT_, CMV-TO:GLUT4_2Ala_, or UBC:DOR_GLUT4(LEYL/AEYA/AEYL/LEAL/LEYA)_-APEX2 alongside pOG44 using Lipofectamine 2000 (Thermo Fisher Scientific, 11668019). Transfected cells were selected with hygromycin at 100 μg/ml (Thermo Fisher Scientific, 10687010) and maintained in hygromycin at 50 μg/ml. For the genome-wide screen, Lenti-X HEK293 cells (Takara Bio, 632180) were transiently transfected with pHR-SFFV-dCas9-BFP-KRAB, pVSVG, and psPAX2 with Lipofectamine 2000. The supernatant was collected after 48 hours and filtered through a 0.45-μm polyethersulfone filter and then incubated overnight with HEK293-FLP cells stably expressing puDNA5-DOR_MRS(WT)_-APEX2. Cells were double sorted for BFP-dCas9 expression and M1-647 MOR expression. Individual clones were isolated and assessed for dCas9 activity. HEK293-FLP cells expressing H1:Sc-shRNA/UBC:SSF-SNAP-MOR_WT_ and H1:VPS35-shRNA/UBC:SSF-SNAP-MOR_WT_ were created by lentiviral transduction as described above. SH-SY5Y cells were purchased from American Type Culture Collection and grown in DMEM with 10% FBS at 37°C and 5% CO_2_. Stable SH-SY5Y cell lines expressing UBC:MOR_WT_-APEX2 were generated using lentiviral transduction as described above. For shRNA experiments in SH-SY5Y cells, SH-SY5Y cells stably expressing UBC:MOR_WT_-APEX2 were further transduced with Scramble shRNA–CMV–green fluorescent protein (GFP) or VPS35 shRNA-CMV-GFP packaged into lentivirus as described above.

### Animals

Timed-pregnant C57BL/6J females were obtained from Charles River Laboratories. All procedures were approved by the Oregon Health and Science University (OHSU) Institutional Animal Care and Use Committee (protocol no. IP00003776) and conducted in accordance with the institutional and federal animal care guidelines.

### Neuronal tissue dissecting and dissociation

Striatal and cortical tissues were isolated from embryonic day 16.5 embryos in ice-cold dissection buffer containing 273.8 mM NaCl, 10 mM KCl, 20 mM NaHCO_3_, 8 mM NaH_2_PO_4_, 1 mM MgSO_4_, 0.5 mM EDTA, 10 mM Hepes, and 25 mM dextrose. Tissue pieces were enzymatically digested in calcium- and magnesium-free Hanks’ balanced salt solution (HBSS; Corning) supplemented with 12 mM MgSO_4_, papain (15 U/ml; Worthington), and deoxyribonuclease I (0.20 mg/ml; Roche) for 25 to 40 min at 37°C. Following digestion, samples were gently triturated using three sequential fire-polished Pasteur pipettes of decreasing diameter to generate single-cell suspensions in calcium- and magnesium-free HBSS.

### Neuronal cell culture

Cell suspensions were counted using a Countess automated cell counter (Thermo Fisher Scientific). Striatal and cortical cells were combined at a 90:10 ratio, and 0.8 × 10^6^ to 1.0 × 10^6^ cells were plated per well onto poly-l-ornithine–coated (0.1 mg/ml) glass-bottom 12-well culture dishes (Cellvis). Cocultures were maintained in modified minimum essential medium (MEM; Gibco; MEM with Earle’s salts and l-glutamine) supplemented with 27.7 mM glucose, 2.38 mM sodium bicarbonate, bovine holo-transferrin (0.10 mg/ml), 5% fetal bovine serum, and 1% B-27 Plus supplement (Thermo Fisher Scientific). Cultures were incubated at 37°C in a humidified atmosphere containing 5% CO_2_. Beginning at day in vitro (DIV) 1, half-volume medium changes were performed every other day.

### OR surface expression, internalization, and recycling

HEK293-FLP cells stably expressing receptor were plated in 12-well plates. Forty-eight hours later, cells were treated with agonist and/or antagonist to determine surface expression, internalization, or recycling. The total condition was treated with 10 μM naloxone for 30 min. The internalization condition was treated with 10 μM agonist (DAMGO for MOR cell lines or DADLE for DOR cell lines) for 30 min. The recycling condition was treated with 30 min of 10 μM agonist followed by a wash with PBS and 30 min of 10 μM naloxone treatment. Cells were washed once with PBS, lifted with TrypLE Express, and resuspended in PBS with calcium and magnesium supplemented with 1% BSA and 1:1000 to 1:2000 M1-647. Cells were labeled with M1-647 for 1 hour at 4°C, then washed once, and resuspended in PBS with calcium and magnesium and 1% BSA. Cells were analyzed using a CytoFLEX S (Beckman Coulter) using the allophycocyanin (APC) channel (638-nm excitation and 660/20-nm emission). Cells were gated for singlets. At least 10,000 singlets were counted for each condition. The geometric mean of the APC channel was used to quantify surface expression of each condition. Internalization was calculated as 1 − (internalization geometric mean/total geometric mean), and recycling was calculated as (recycling geometric mean − internalization geometric mean)/(total geometric mean − internalization geometric mean). Total surface expression was calculated as the total geometric mean divided by the geometric mean of a labeled nonexpressing parental control. We ensured that surface receptor labeling in all stably expressing lines was sufficiently above background so that potential contributions from background in the internalization calculations were negligible.

SH-SY5Y cells were handled the same way but, after ligand treatment and lifting, were resuspended in PBS with calcium and magnesium supplemented with 1% BSA and 1:1000 M1 anti-FLAG and incubated for 1 hour at 4°C. Cells were then washed and resuspended in PBS with calcium and magnesium supplemented with 1% BSA and 1:1000 goat anti-mouse 647, then washed once, and prepared for flow cytometry measurements as described for HEK293-FLP cells. For cells transduced with shRNA, cells were additionally gated for expression of GFP, and 10,000 cells expressing GFP were counted for each condition. Calculations for trafficking were performed as described above for HEK293-FLP cells.

### cAMP signaling assay

HEK293-FLP cells were plated at 20% confluency in six-well plates. The following day, cells were transfected with 1 μg of receptor [UBC:MOR_WT_, MOR_2Ala_, DOR_WT_, DOR_MRS(WT)_, DOR_MRS(2Ala)_, or DOR_GLUT4(LEYL)_], 2.5 μg of GloSensor20F-IRES-Rluc, and 9.6 μl of Lipofectamine 2000. Twenty-four hours after transfection, cells were lifted, resuspended in 30 mM Hepes in DMEM without phenol red (Gibco, A1896702) with 1.6 mM d-luciferin (LUCNA, GoldBio), and incubated for 1 hour at 37°C and 5% CO_2_. Following incubation, cells were plated into white 96-well half-volume plates and treated with 30 nM isoproterenol [to stimulate cyclic adenosine 3′,5′-monophosphate (cAMP) production], 30 nM isoproterenol and 10 μM DAMGO or DADLE, or 30 nM isoproterenol and 500 μM IBMX (Cell Signaling Technology, 13630) (to verify lack of sensor saturation) made up in 30 mM Hepes in DMEM without phenol red with 1.6 mM d-luciferin. To determine the baseline, some cells were treated with only 30 mM Hepes in DMEM without phenol red with 1.6 mM d-luciferin with no added drugs. Each condition was performed in triplicate. Luminescence counts were read using the luminescence module on a Spark multimode microplate reader (Tecan) at 37°C across 10 to 20 min. Cells were then treated with a buffer containing 25 mM Hepes (pH 7.4), 1 mM EDTA, 0.4 mM DTT, 0.2% Triton X-100, and 10 μM coelenterazine h (Thermo Fisher Scientific, C6780), which eliminates luciferase luminescence and allows for normalization of potential differences in cell number plating and transfection levels. Luminescence was then read for an additional 5 min. To calculate cAMP inhibition, luminescence values were normalized and background subtracted. The normalized technical triplicates were averaged, and the averages of the highest three values for each condition were used to calculate percent cAMP inhibition using the following formula: [1 − (isoproterenol + opioid agonist)/soproterenol only] × 100.

### AMPLEX assay, lysate

HEK293-FLP cells stably expressing receptor were plated in 24-well plates. Forty-eight hours later, cells were stimulated with 10 μM agonist (DAMGO or DADLE) for the indicated duration in the figure legends. Experiments were performed in technical duplicate. Cells were then lysed for 3 min in ice-cold PBS with 0.1% Triton X-100. After lysis, PBS with 0.1% Triton X-100, 100 μM AUR, and 200 μM H_2_O_2_ was added to the cells to provide the necessary substrates for the AMPLEX reaction. Two minutes later, the reaction was stopped with PBS with 30 mM sodium ascorbate. Fluorescence intensity was measured at 561-nm excitation and 610-nm emission on a Spark multimode microplate reader (Tecan). Percent GPCR-APEX2 was calculated as the amount of fluorescence after agonist treatment divided by the amount of fluorescence without agonist treatment multiplied by 100%. For experiments using bafilomycin A, cells were treated with 100 nM bafilomycin A for 3 hours before agonist treatment. DAMGO (10 μM) was then added directly to wells for the noted times. Control wells were treated with DMSO vehicle (0.1%).

### AMPLEX assay, intact cells

HEK293-FLP cells stably expressing puDNA5-DOR_MRS(WT)_-APEX2 were plated in 24-well plates. Forty-eight hours later, cells were treated with agonist for 2 hours and 15 min or left untreated. Cells were then lifted with TrypLE Express and pelleted with DMEM and 10% FBS. Cells were resuspended in ice-cold PBS with 200 μM AUR and incubated for 5 min at room temperature and then for 10 min on ice. Next, PBS with 4% (w/v) BSA and 100 μM H_2_O_2_ was added to the cells. Thirty seconds later, the reaction was quenched with 1 mM sodium azide. Cells were then washed with PBS with 2% BSA and resuspended in PBS with 1% BSA. Cells were analyzed on a CytoFLEX S using the APC channel.

### Genome-wide CRISPRi screen

HEK293-FLP cells expressing puDNA5-DOR_MRS(WT)_-APEX2 and dCas9-BFP-KRAB were transduced with three different CRISPRi sgRNA sublibraries to obtain coverage of the entire human genome. Transduced cells were selected for with puromycin (0.75 μg/ml; Gibco, A1113803) 48 hours after transduction. Six days after transduction, the intact cell GPCR-APEX/AUR assay was performed. All samples were pooled and passed through a 40-μm filter and then analyzed on a BD FACSAria II. Cells were gated for singlets and then for BFP positive and then sorted into the top and bottom quartiles using the APC channel. DNA from these cells was collected with QIAamp DNA Blood Mini kits (QIAGEN, 51104). sgRNA libraries were prepared using Q5 Hot Start High-Fidelity DNA Polymerase (NEB, M0493L) and barcoding primers. PCR products were purified using QIAquick PCR purification columns (QIAGEN, 28106) and loaded on 20% Tris-borate-EDTA (TBE) gels (Thermo Fisher Scientific, EC63155BOX). A 270–base pair (bp) gel was excited from the gel and quantified using a Bioanalyzer (Agilent) and sequenced on an Illumina HiSeq 4000 system (Illumina) using custom primers.

### Bioinformatic analysis of CRISPRi screen and comparison to DOR_WT_-APEX2 screen

Deconvoluted reads from each sublibrary were downloaded from Novogene, aligned to the start of the sgRNAs, and cropped to 38 bp long. Cropped reads for each sublibrary were then loaded into ScreenProcessing (https://doi.org/10.5281/zenodo.17849246 from https://github.com/mhorlbeck/ScreenProcessing) python script to count reads of each guide, compute effect sizes by comparing top and bottom quartiles, and compile data from multiple individual guides for each gene to compute an average effect and Mann-Whitney *P* value of the gene. From negative control guides, pseudogenes were assembled, and hits were determined by thresholding effect and *P* value such that less than 10% of hits were pseudogenes. Genome-wide screen data were deposited to the Gene Expression Omnibus (accession code GSE283358).

### Analysis of mouse brain dataset for expression of CRISPRi hits

Single-nucleus RNA sequencing data for each of the 146 hits from the CRISPRi screen, as well as the MOR, were downloaded for each cell metacluster from www.braincelldata.org (*115*). The average percent expression of each hit gene across the top 10 MOR-expressing neuron metaclusters was calculated, and hits were considered expressed in MOR neurons if their average expression was greater than 10%. If a hit was not identified within the dataset, then its expression was considered to be 0.

### siRNA transfections

All siRNAs were purchased from Dharmacon-Horizon Discovery and resuspended in ribonuclease-free water (B-003000-WB) according to the manufacturer’s protocols. The following siRNAs were used: nontargeting control pool (NTC; NC1486135), VPS35 pool (L-010894-00) as well as the four individual siRNAs that make up that pool, VPS29 pool (L-009764-001), VPS26A pool (L-013195-00), ARF6 pool (L-004008-00), VPS35L pool (L-018658-02), SNX3 pool (L-011521-01), and CLTC pool (L-004001-01). siRNA transfections were performed as “reverse” transfections. siRNA (100 pmol) and DharmaFECT 1 (17 μl; T-2001-03, Dharmacon) were incubated for 20 min in Opti-MEM (Thermo Fisher Scientific, 31985070), then added to a cell suspension, and seeded at 40% confluency in a T25 cell culture flask. After 24 hours, cells were split into plates for trafficking experiments. Experiments were conducted 72 hours after transfection.

### Western blot

Cells were lysed in-well with ice-cold radioimmunoprecipitation assay (RIPA) buffer [50 mM tris, 150 mM NaCl, 1% Triton X-100, 0.5% sodium deoxycholate, and 0.1% SDS (pH 7.4)]. Halt Protease Inhibitor Cocktail (Thermo Fisher Scientific, 78430) was added to the RIPA buffer immediately before use. Cell lysates were incubated on ice for 10 min, sonicated, and centrifuged. The supernatant was added to sample loading buffer with 1% (v/v) 2-mercaptoethanol (Bio-Rad, 1610710). Samples were boiled at 95°C for 5 min. Proteins were then separated on a Bio-Rad 4 to 20% Mini-PROTEAN TGX Stain-Free Protein Gel (4568096 or 4568095) in SDS–polyacrylamide gel electrophoresis running buffer (0.2501 M tris, 1.924 M glycine, and 0.0347 M SDS). Gels were stain-free activated using a Bio-Rad ChemiDoc Imaging System and transferred to nitrocellulose membranes. Membranes were blocked in Bio-Rad Everyblot Blocking Buffer (12010020) for 1 hour at room temperature and then incubated with primary antibody overnight at 4°C. Blots were washed four times with PBS with 0.1% (v/v) Tween, incubated with secondary antibody for 1 hour at room temperature, and then washed four more times. Blots were imaged on a Bio-Rad ChemiDoc Imaging System. Contrast and brightness were adjusted across the entire uncropped blot using ImageJ-Fiji. Proteins were quantified by normalizing the intensity of the indicated band to the stain-free protein loading control for each lane and then normalizing the indicated protein expression in the control sample to 100% to evaluate knockdown.

### Small hairpin RNA transduction

HEK293-FLP cells were transduced with H1:Sc-shRNA/UBC:SSF-SNAP-MOR_WT_ or H1:VPS35-shRNA/UBC:SSF-SNAP-MOR_WT_ packaged into lentivirus as described in the cell culture section. Transduced cells were used in experiments at least 1 week after transduction. SH-SY5Y cells stably expressing puDNA5-MOR_WT_-APEX2 were transduced with Scramble shRNA-CMV-GFP or VPS35 shRNA-CMV-GFP packaged into lentivirus using LentiX HEK293 cells as described in the cell culture section. Transduced SH-SY5Y cells were used in experiments at least 5 days after transduction to ensure sufficient knockdown and used for up to five passages. Neurons were transduced between DIV 7 and DIV 21 with H1:Sc-shRNA/UBC:SSF-SNAP-MOR_WT_ or H1:VPS35-shRNA/UBC:SSF-SNAP-MOR_WT_ packaged into lentivirus as described in the cell culture section. Viral particles were applied at a multiplicity of infection of 0.5 to 1.0, adjusted according to downstream assay requirements. Cultures were maintained for an additional 7 days posttransduction to allow robust transgene expression.

### Fixed imaging sample preparation

For analysis of MOR delivery to lysosomes, HEK293-FLP cells transduced with H1:Sc-shRNA/UBC:SSF-SNAP-MOR_WT_ or H1:VPS35-shRNA/UBC:SSF-SNAP-MOR_WT_ were plated onto poly-l-lysine (Sigma-Aldrich, P8920)–coated coverslips in 24-well plates. Forty-eight hours later, the cells were labeled with 30 nM SNAP-JF549 for 30 min at 37°C, washed twice with PBS, and then treated with 10 μM DAMGO for the indicated times. Following agonist treatment, cells were then fixed with PBS with 4% (v/v) paraformaldehyde for 20 min at room temperature, washed twice with PBS with calcium and magnesium, and blocked and permeabilized with imaging buffer [PBS with calcium and magnesium, 3% (w/v) BSA, and 0.1% (v/v) Triton X-100] for 1 hour at room temperature. Cells were then incubated with anti-LAMP antibody (1:200) in fresh imaging buffer overnight at 4°C. Cells were washed twice with PBS with calcium and magnesium and then incubated with secondary antibody (1:1000) in imaging buffer for 1 hour at room temperature. Cells were washed twice with PBS with calcium and magnesium, then mounted on glass slides with ProLong Diamond mounting medium (P36962), and dried overnight. Samples were imaged on a Zeiss LSM 900 with Airyscan 2 with a 63× Oil 1.4 numerical aperture (NA) Plan-Apo lens and Airyscan processed in ZEN Blue software (Zeiss). Single confocal slice images were processed in ImageJ-Fiji.

For analysis of spatial relationship between MOR and Retromer, HEK293-FLP cells or SH-SY5Y cells stably expressing puDNA5-MOR_WT_-APEX2 were plated onto poly-l-lysine (Sigma-Aldrich, P8920)–coated coverslips in 24-well plates. Forty-eight hours later, cells were incubated with M1 anti-FLAG (1:500) for 30 min, washed once with PBS with calcium and magnesium, and then treated with 10 μM DAMGO for 20 min. Cells were then fixed and prepared as described above, except the primary antibodies used overnight were anti-VPS35 (1:500) and anti-GM130 (1:500).

### Imaris-based analysis of fixed imaging experiments: Percent object overlap, object proximity, and Pearson’s

Airyscan images were converted using Imaris File Converter, and 3D surfaces for each channel were produced using the Surfaces tool in Imaris (v10). Surface grain size was set to 0.4 μm. Each individual surface object representing an endocytosed receptor (561-nm channel for LAMP1 and MOR overlap experiments and 647 channel for VPS35/Golgi and MOR proximity experiments) was analyzed for its closest proximity to VPS35 or Golgi objects in the 488-nm channel (for VPS35/Golgi and MOR experiments) or percentage overlap with LAMP1-positive surfaces (647-nm channel for LAMP1 and MOR experiments). Three full *z*-stacks from one slide (technical replicates) were analyzed for three independently prepared slides (biological replicates). Results are displayed as the average percent overlap value across the technical replicates for three biological replicates (Fig. 3H), as the nearest proximity between all MOR objects and a labeled protein object (Fig. 4B and fig. S5D), or as the average median value of the nearest proximity across three technical replicates for three biological replicates (Fig. 4C and fig. S5E). Pearson’s correlation coefficient was calculated in Imaris software from three *z*-stacks per slide (technical replicates) and three independently prepared slides per condition (biological replicates). Images were masked for the 647 channel (MOR) and automatic thresholding was performed. Each Pearson’s coefficient value displayed represents the average value of three technical replicates.

### RNA fluorescence in situ hybridization (HCR-FISH)

RNA FISH was performed using hybridization chain reaction (HCR) v3 reagents (Molecular Instruments) according to the manufacturer’s protocol. Cocultures were fixed in 4% paraformaldehyde at room temperature for 30 min, followed by processing in the HCR v3 buffer system. A probe set targeting *Mus musculus Adora2a* mRNA (B2 amplifier; Molecular Instruments) was applied and hybridized overnight at 37°C. Signal amplification was performed using Alexa Fluor 488–conjugated B2 hairpins. Nuclei were counterstained with 4′,6-diamidino-2-phenylindole (1:10,000 dilution; Thermo Fisher Scientific) during the final wash step. Fluorescence images were acquired on a Zeiss LSM 980 confocal microscope using a 20× objective, with identical laser power and acquisition settings maintained across all experimental conditions to ensure comparability. Bright-field images were acquired on a Zeiss Celldiscoverer 7 using the Plan-Apochromat 5×/0.35 objective in combination with the internal 2× optical zoom module.

### Live imaging of primary striatal neurons

Seven to 8 days after transduction, neurons expressing H1:Sc-shRNA/UBC:SSF-SNAP-MOR_WT_ or H1:VPS35-shRNA/UBC:SSF-SNAP-MOR_WT_ were labeled with 1 μM SNAP-JF549 for 15 min at 37°C. Neurons were washed once with PBS and then supplemented with preconditioned medium. The plate was then moved to the imaging incubator on a Nikon spinning-disk confocal microscope (Yokogawa CSU-W1). Cells were imaged with a 60× Oil 1.4 NA Plan-Apo lens using the 488-nm laser (to image GFP) and the 561-nm laser (to image SNAP-JF549). Wells were then treated for 30 min with 1 μM DAMGO. After this, medium was removed and replaced with preconditioned medium containing 10 μM naloxone. Images of each neuron were acquired before the start of treatment, after agonist treatment, and after antagonist treatment using identical laser power and acquisition settings across all experimental conditions. For experiments using SNAP-Surface Block, cells were prelabeled with 1 μM SNAP-Surface Block for 15 min at 37°C. Neurons were washed once with PBS and then labeled with 1 μM SNAP-JF549 as described above. Samples for all neuron experiments were collected from at least two independent culture preparations, and 8 to 13 neurons per condition were imaged in each imaging session.

### Analysis of neuron imaging

Before analysis, images were grouped by individual neuron and blinded for the shRNA condition. Images were processed in Fiji-ImageJ by drawing regions of interest around the cell membrane of a neuron. This allowed for splitting of signal into surface and internal signal and removal of signal outside the neuron. For each neuron, the intensity of SNAP-JF549 signal on the surface and across the whole cell was measured and used to create a surface fluorescence ratio by dividing the amount of fluorescence on the cell surface with the total amount in the cell. Recycling was calculated as the difference between the surface ratio after antagonist treatment and before antagonist treatment. Internalization was calculated as the difference between the surface ratio after agonist treatment and before agonist treatment.

### Bioinformatics

The entry identifiers for all Swiss-Prot–reviewed human proteins tagged with the keyword “Cell Membrane” (KW-1003) were downloaded from the UniProt Knowledgebase. This list of 4019 proteins was uploaded into our custom MotifSearcher application (https://doi.org/10.5281/zenodo.17845942) and searched for proteins where the last amino acid in the sequence was within a region with a topological domain annotated (either cytoplasmic or extracellular). This list was downloaded into Microsoft Excel and filtered to exclude proteins with an extracellular annotation resulting in a final list of 2359 proteins with cytoplasmic tails. All remaining searches were performed in this list. The following searches were conducted in the last 100 amino acids of the protein: “L@@L” (to identify LxxL motifs), “N@@Y” (to identify N[P/x]xY motifs), “[FYW]@[LMV]” (to identify [F/Y/W]x[L/M/V] motifs), and “[GAVCPLIMWF]@[FYV]@[FY]” (to identify Φx[F/Y/V]x[F/Y] motifs), where @ is any amino acid, and any of the bracketed amino acids are accepted within the defined position in the sequence. The PDZ binding motif [D/E][S/T]@Φ-COOH, “[DE][ST]@[GAVCPLIMWF],” was searched in the last four amino acids of each protein to ensure that it encompassed the C terminus. The resulting lists were downloaded into Microsoft Excel and filtered to exclude motifs found in extracellular regions as well as duplicate proteins from instances where a motif was found multiple times. The final list of unique proteins for each motif was categorized for protein class using the PANTHER database (*116*). For LxxL motifs, the list was further parsed on the basis of whether the LxxL motif was found at least 15 amino acids away from the plasma membrane.

### GLUT4 surface expression

HEK293-TREx cells expressing GLUT4(WT) and GLUT4(2Ala) were reverse transfected with either NTC, CLTC, or VPS35 siRNA and plated in 12-well plates at 10% confluency. The following day, GLUT4 expression was induced by replacing the medium with medium containing doxycycline (10 ng/ml). Cells were washed once with PBS and supplemented with fresh medium lacking doxycycline 4 hours later. Sixteen hours later, cells were lifted and labeled with 1:1000 anti-myc for 1 hour at 4°C, washed once, and then labeled with 1:1000 goat anti-mouse 647 for 1 hour. Cells were then prepared for flow cytometry measurement as described in the OR surface expression section for HEK293-FLP and SH-SY5Y cells. Surface expression was calculated by dividing the geometric means of the APC channel for each induced condition and an uninduced control.

### Statistical analysis and reproducibility

Statistical analysis was performed in Prism (GraphPad) or published software for genomics (ScreenProcessing_v0.1). For immortalized cell line experiments, all experiments except the genome-wide screen include results from at least three biological replicates. Representative flow cytometry histograms, Western blots, or confocal images are from at least three biological replicates. Plotted data are represented as individual biological replicates or as the mean of at least three biological replicates ± SD, except for Fig. 4B and fig. S5D, which include all technical replicates from three biological replicates. The genome-wide screen was performed once across three independent sublibraries. For neuron experiments, all experiments were conducted from at least two independent preparations. Figure S6F plots all technical replicates from two independent imaging sessions. Figure 5G and fig. S6E are superplots that show all technical replicates from six independent biological replicates, as well as medians from each biological replicate (defined as images taken from one independent well of cells). In these figures, statistical tests are performed on the medians (*n* = 6).

All measurements were taken from distinct samples, with the exception of the NTC internalization data in Fig. 6C, which replots data from Fig. 6B for analysis display purposes. These data were collected as part of the same experiment. Figure S7F replots DOR_WT_ data from fig. S1J; these data were collected as part of the same experiment but are plotted in separate figures for narrative clarity. *P* values are represented as not significant (n.s.) if *P* > 0.05, * if *P* ≤ 0.05, ** if *P* ≤ 0.01, *** if *P* ≤ 0.001, and **** if *P* ≤ 0.0001. In Fig. 3E, some *P* values are represented as #’s instead of *’s to differentiate between different comparisons.

### Software and code

Data were collected with the following software: flow cytometry (Beckman CytExpert v2.4), plate reader (Tecan Spark Control v3), Western blot (Bio-Rad Image Lab Touch v2.4 and Fiji-ImageJ v1.54f), and microscopy (ZEN v3.5). Data were analyzed with the following software: statistical analysis and graphing (GraphPad Prism v10), flow cytometry (FlowJo v9 or v10), genome-wide screen (open-source custom software ScreenProcessing_v0.1 and RStudio v2023.09.01 build 494), and microscopy (Imaris v10, Fiji-ImageJ v1.54f). The version of the custom code for the MotifSearcher application used in the bioinformatics searches is available on GitHub (https://doi.org/10.5281/zenodo.17845942).
