# Supplementary material for: Lysosomal down-regulation of the mu opioid receptor is opposed by the Retromer complex
Source: Sci Adv. 2026 Mar 20;12(12):eadx8715. doi: 10.1126/sciadv.adx8715 (PMC13004025; doi:10.1126/sciadv.adx8715)
Supplement: Supplementary file 1 — Figs. S1 to S7 Legends for tables S1 to S4 [file sciadv.adx8715_sm.pdf]

Science Advances
AAAS

Supplementary Materials for

Lysosomal down-regulation of the mu opioid receptor is opposed by the Retromer complex

Aleksandra Dagunts *et al.*

Corresponding author: Braden T. Lobingier, lobingib@ohsu.edu

Sci. Adv. **12**, eadx8715 (2026)
DOI: 10.1126/sciadv.adx8715

The PDF file includes:

Figs. S1 to S7
Legends for tables S1 to S4

Other Supplementary Material for this manuscript includes the following:

Tables S1 to S4

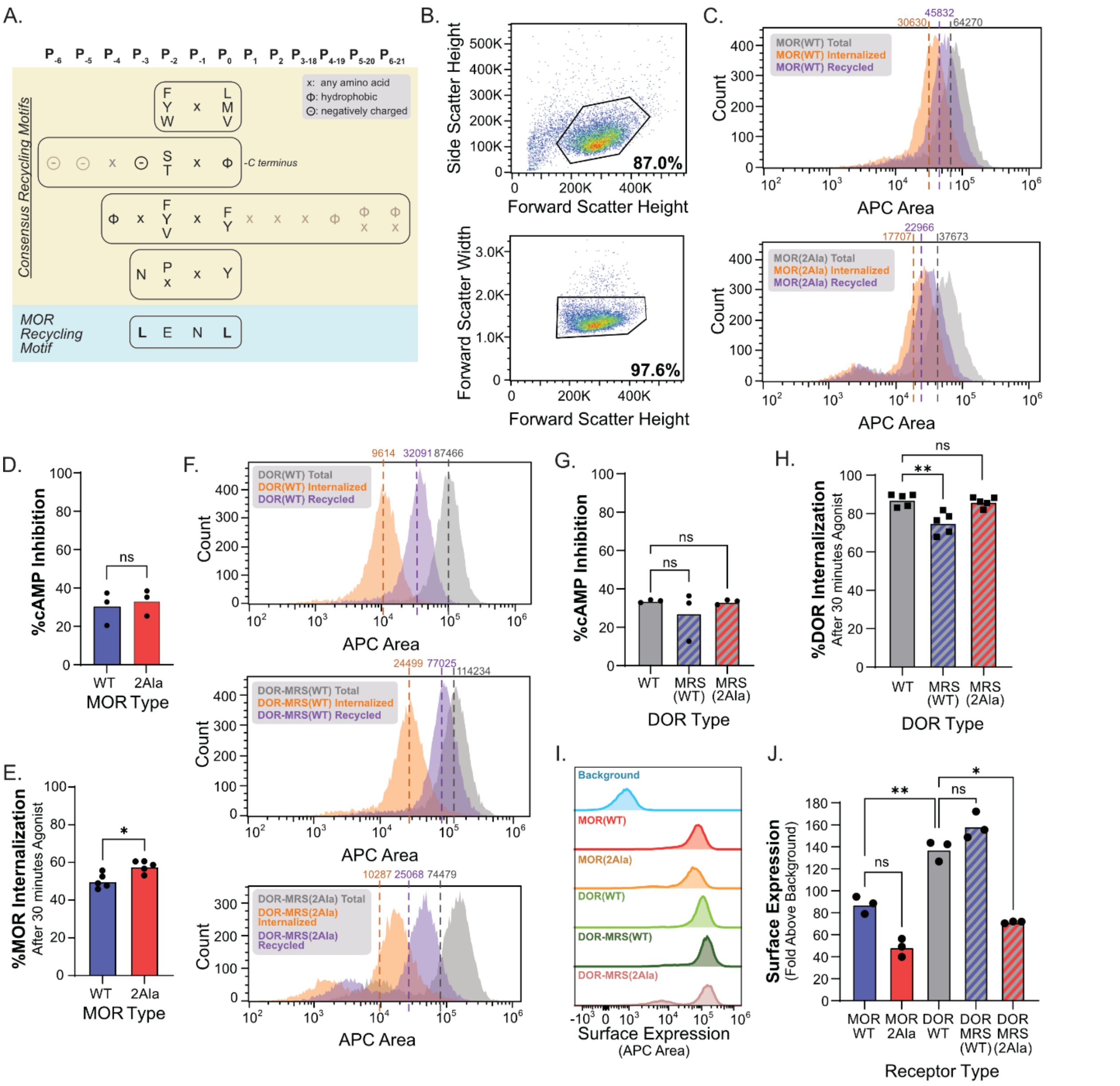

Fig. S1. The GPCR-APEX2 downregulation assay captures changes in GPCR endosomal recycling. A. Comparison of known recycling motifs and the MOR recycling motif. Complete sequences are shown in tan, and more minimal motif amino acid sequences referenced throughout the text are shown in black. **B.** Example gating scheme for single HEK293-FLP cells in flow cytometry-based assays. Cells are gated to exclude debris (top) then gated to exclude doublets (bottom). All subsequent analysis is done on this population. **C.** Example histograms of MOR(WT) (top) and MOR(2Ala) (bottom) for stably expressing HEK293-FLP cells treated with 10 µM naloxone for 30 minutes (total), 10 µM DAMGO for 30 minutes (internalized), or 10 µM DAMGO for 30 minutes followed by 10 µM naloxone for 30 minutes (recycled). Geometric means for each curve are noted. **D.** Percent cAMP inhibition in HEK293-FLP cells transiently expressing MOR(WT) or MOR(2Ala) over 20 minutes of treatment with 10 µM DAMGO and 30 nM isoproterenol (n=3, two-tailed paired t-test, p=0.37). **E.** Percent MOR internalization in

stably expressing HEK293-FLP cells after 30 minutes of 10 µM DAMGO treatment (n=5, two-tailed paired t-test, p=0.025). **F.** Example histograms of DOR(WT) (top), DOR-MRS(WT) (middle), and DOR-MRS(2Ala) (bottom) for stably expressing HEK293-FLP cells treated with 10 µM naloxone for 30 minutes (total), 10 µM DAMGO for 30 minutes (internalized), or 10 µM DAMGO for 30 minutes followed by 10 µM naloxone for 30 minutes (recycled). Geometric means for each curve are noted. **G**. Percent cAMP inhibition in HEK293-FLP cells transiently expressing DOR(WT), DOR-MRS(WT), or DOR-MRS(2Ala) over 20 minutes of treatment with 10 µM DADLE and 30 nM isoproterenol (n=3, one-way RM ANOVA, p=0.63 and 0.40 for DOR(WT) vs DOR-MRS(WT) and DOR-MRS(2Ala) respectively). **H.** Percent DOR internalization in stably expressing HEK293-FLP cells after 30 minutes of 10 µM DADLE treatment (n=5, one-way RM ANOVA, p=0.0012 and 0.31 for DOR(WT) vs. DOR-MRS(WT) and DOR-MRS(2Ala) respectively). **I.** Example histogram of surface anti-FLAG staining of receptors in stably expressing HEK293-FLP lines compared to non-expressing parental HEK293-FLP cells (background). **J.** Quantification of antibody detection of surface receptor expression in HEK293-FLP cells compared to background antibody staining (n=3, one-way RM ANOVA, p=0.075 and 0.0092 for MOR(WT) vs MOR(2Ala) and DOR(WT) respectively, p=0.20 and 0.022 for DOR(WT) vs. DOR-MRS(WT) and DOR-MRS(2Ala) respectively).

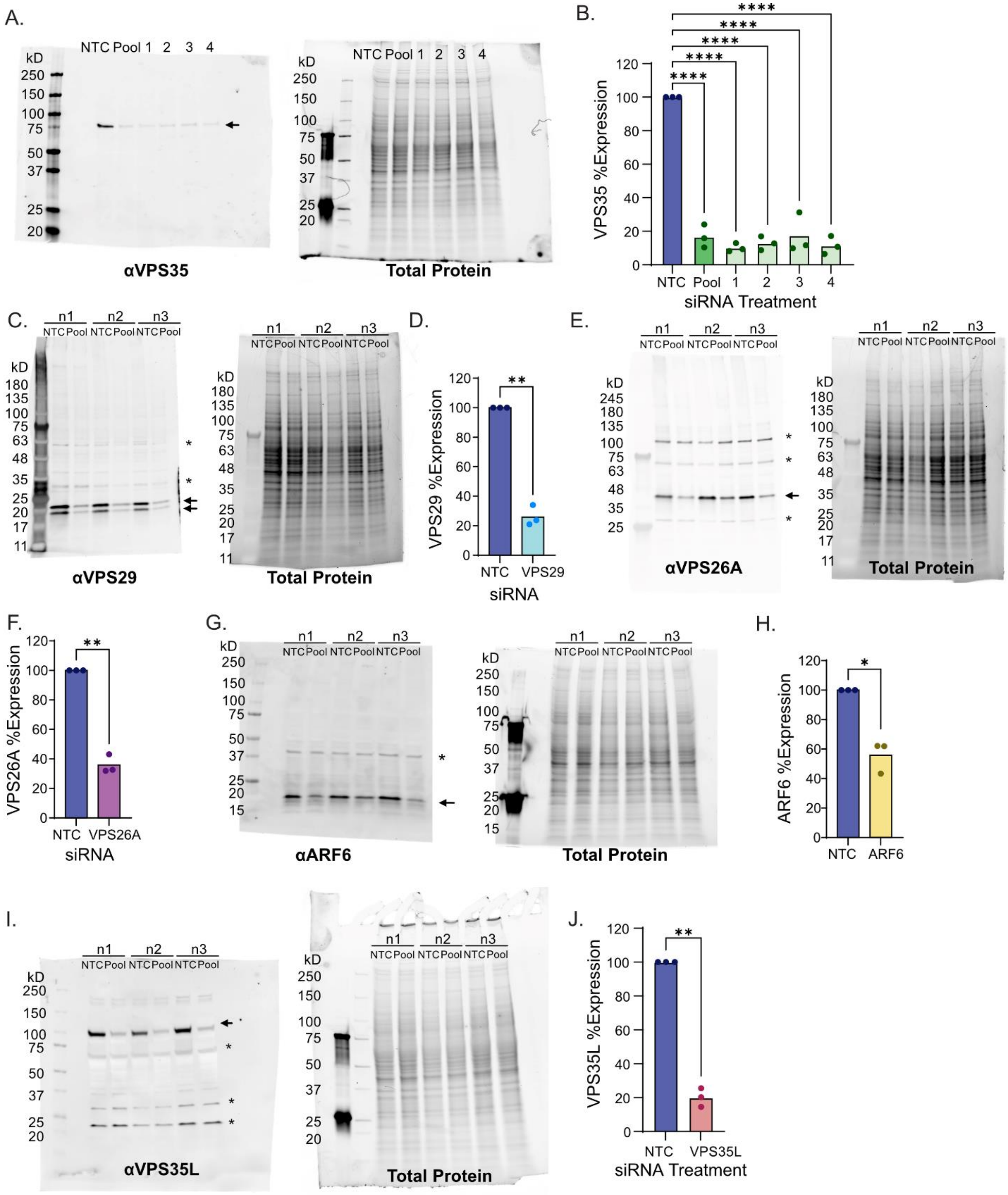

Fig. S2. The LENL motif requires Retromer to oppose agonist-induced opioid receptor downregulation. A. Western blot of HEK293-FLP cells stably expressing MOR(WT) treated with either NTC siRNA or pooled or individual siRNAs against VPS35. Representative western blot shown from n=3. Arrow denotes VPS35. **B.** Quantification of siRNA knock-down of VPS35 normalized for total protein and to NTC expression (n=3, one-way RM ANOVA with Dunnett's

multiple comparisons correction, p<0.0001 for NTC vs. Pool, 1, 2, 3, and 4). **C.** Western blot of HEK293-FLP cells stably expressing MOR(WT) treated with either NTC siRNA or pooled siRNAs against VPS29. All three replicates shown. Arrow denotes VPS29. Asterisks denote off-target bands. **D.** Quantification of siRNA knock-down of VPS29 normalized for total protein and to NTC expression (n=3, two-tailed paired t-test, p=0.0029). **E.** Western blot of HEK293-FLP cells stably expressing MOR(WT) treated with either NTC siRNA or pooled siRNAs against VPS26A. All three replicates shown. Arrows denotes VPS29. Asterisks denote off-target bands. **F.** Quantification of siRNA knock-down of VPS26A normalized for total protein and to NTC expression (n=3, two-tailed paired t-test, p=0.0032). **G.** Western blot of HEK293-FLP cells stably expressing DOR-MRS(WT) treated with either NTC siRNA or pooled siRNAs against ARF6. All three replicates shown. Arrow denotes ARF6. Asterisks denote off-target bands. **H.** Quantification of siRNA knock-down of ARF6 normalized for total protein and to NTC expression (n=3, two-tailed paired t-test, p=0.0194). **I.** Western blot of HEK293-FLP cells stably expressing MOR(WT) treated with either NTC siRNA or pooled siRNAs against VPS35L. All three replicates shown. Arrow denotes VPS35L. Asterisks denote off-target bands. **J.** Quantification of siRNA knock-down of VPS35L normalized for total protein and to NTC expression (n=3, two-tailed paired t-test, p=0.0016).

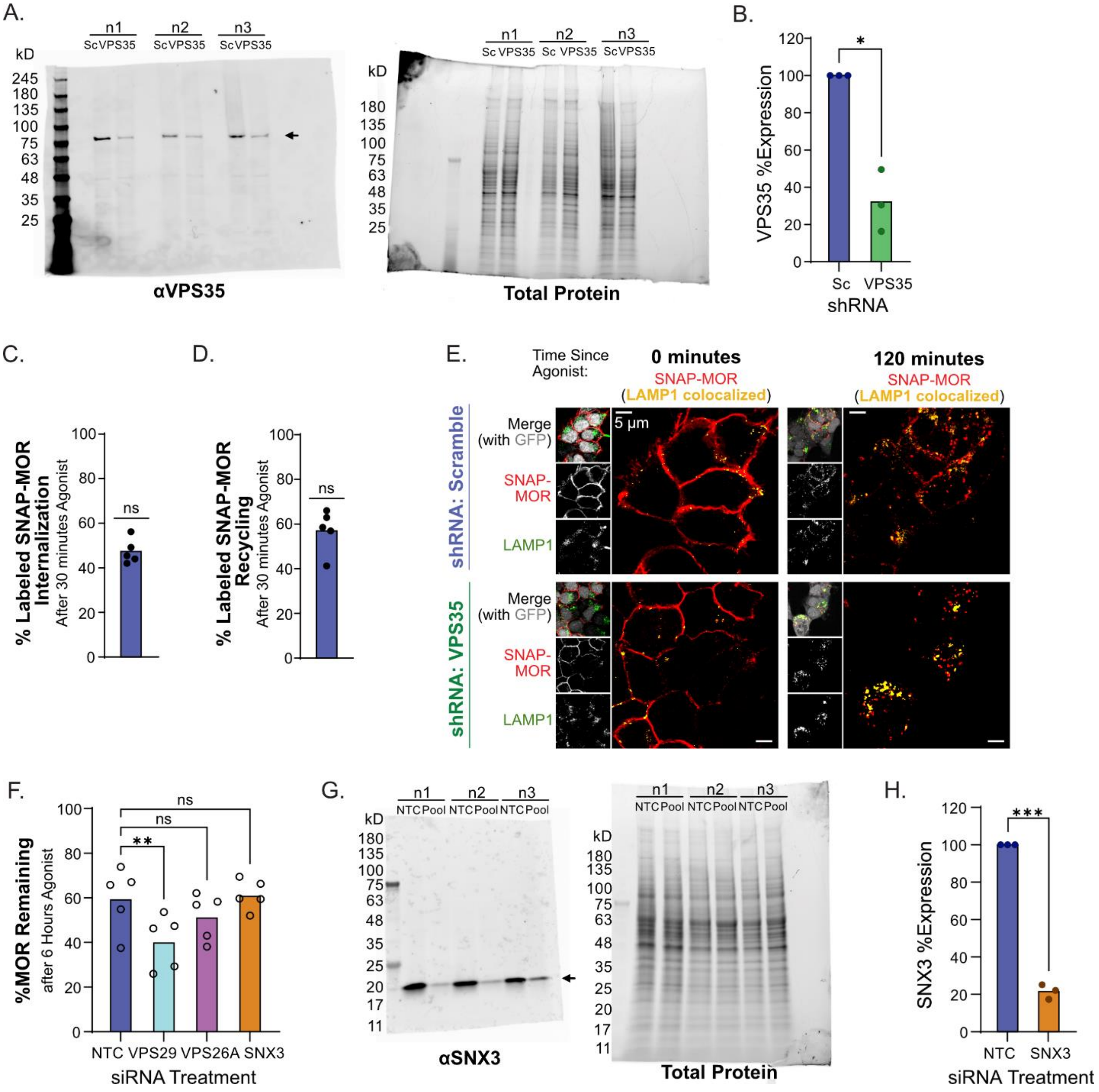

Fig. S3. **Retromer functions through the bileucine LENL motif to protect MOR from lysosomal downregulation. A**. Uncropped western blot of HEK293-FLP cells stably expressing Sc or VPS35/SNAP-MOR. All three replicates shown. Arrow denotes VPS35. **B.** Quantification of shRNA knock-down of VPS35 normalized for total protein and to Sc VPS35 expression (n=3, two-tailed paired t-test, p=0.020). **C.** Percent internalization of SNAP-MOR in HEK293-FLP cells expressing Sc/SNAP-MOR after 30 minutes of labeling with 30 nM SNAP-J549 followed by 30 minutes of 10 µM DAMGO treatment (n=5, one-sample t-test against 49.99, the mean of MOR(WT) internalization in Supplemental Figure 1D, p=0.36). **D.** Percent recycling of SNAP-MOR in HEK293-FLP cells expressing Sc/SNAP-MOR after 30 minutes of labeling with 30 nM SNAP-J549 followed by 30 minutes of 10 µM DAMGO treatment and 30 minutes 10 µM naloxone treatment (n=5, one-sample t-test against 57.812, the mean of MOR(WT) recycling in Figure 1B, p=0.89). **E.** Additional example of confocal images as described for Figure 3G from

n=3 independent experiments. **F.** Percent MOR(WT) remaining in stably expressing HEK293-FLP cells following siRNA knock-down of VPS29, VPS26A, or SNX3 and treatment with 6 hours of 10 μM DAMGO followed by the APEX2/AUR reaction, normalized to no agonist treatment (n=5, one-way RM ANOVA with Dunnett's multiple comparisons correction, p=0.0024, 0.0549, 0.9820 for NTC vs VPS29, VPS26A, and SNX3 respectively). **G.** Western blot of HEK293-FLP cells stably expressing MOR(WT) treated with either NTC siRNA or pooled siRNAs against SNX3. All three replicates shown. Arrow denotes SNX3. **H.** Quantification of siRNA knock-down of SNX3 normalized for total protein and to NTC expression (n=3, two-tailed paired t-test, p=0.00090).

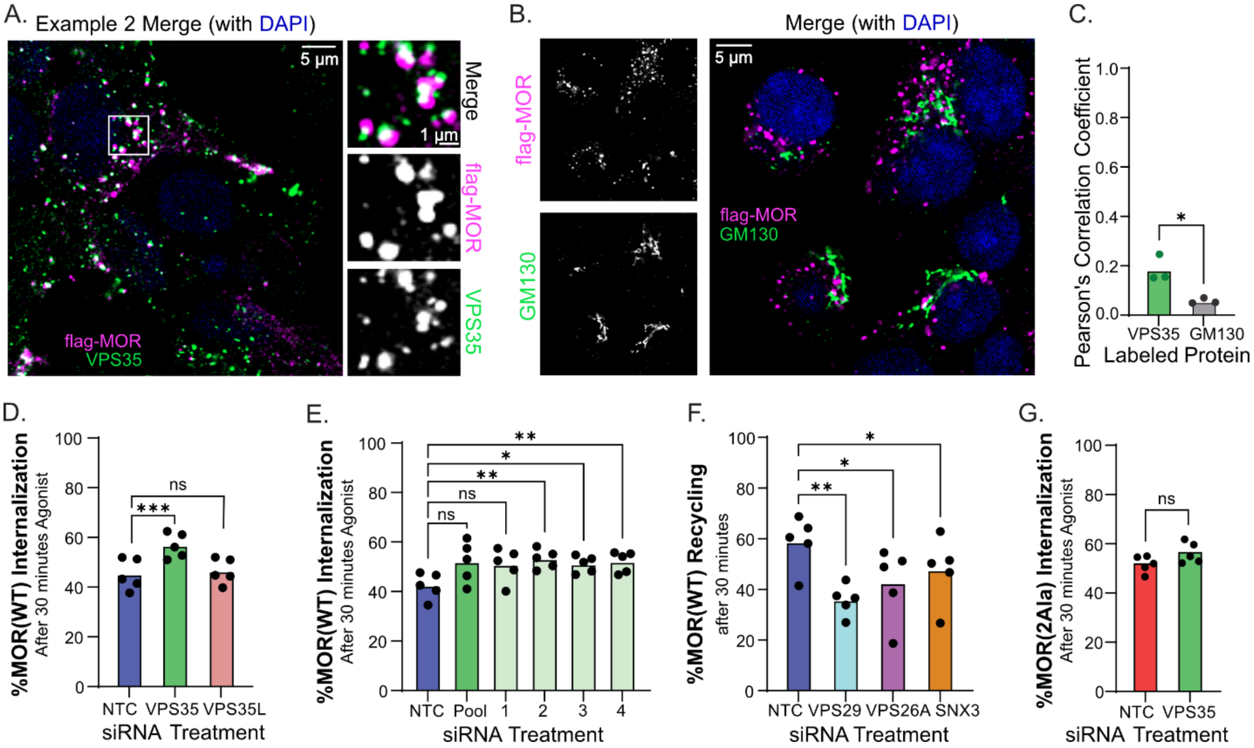

Fig. S4. The bileucine LENL motif gives MOR access to the Retromer-mediated endosomal recycling pathway. A. Additional example of MOR(WT) and VPS35 confocal imaging as described in Figure 4A. B. Example of MOR(WT) confocal imaging as described in Figure 4A but stained for anti-GM130 (green) instead of anti-VPS35 following fixation. **C.** Pearson's correlation coefficient for co-localization of MOR(WT) and VPS35 or GM130 (n=3, two-tailed unpaired t-test, p=0.016). **D.** Percent MOR(WT) internalization in stably expressing HEK293-FLP cells treated with siRNAs against VPS35 or VPS35L following 30 minutes of treatment with 10 µM DAMGO measured by surface receptor staining (n=5, one-way RM ANOVA with Dunnett's multiple comparisons correction, p=0.00070 and 0.65 for NTC vs. VPS35 and VPS35L respectively). **E.** Same as D, but MOR(WT) HEK293-FLP cells were treated with individual or pooled siRNAs against VPS35 (n=5, one-way RM ANOVA with Dunnett's multiple comparisons correction, p=0.051, 0.11, 0.0066, 0.016, 0.0060 for NTC vs. Pool, 1, 2, 3, and 4 respectively). **F.** Percent MOR(WT) recycling as described in Figure 4D, but in HEK293-FLP cells treated with siRNAs against VPS29, VPS36A, or SNX3 (n=5, one-way RM ANOVA with Dunnett's multiple comparisons correction, p=0.0070, 0.011, 0.018 for NTC vs VPS29, VPS26A, and SNX3 respectively). **G.** Percent MOR(2Ala) internalization in stably expressing HEK293-FLP cells treated with siRNAs against VPS35 after 30 minutes of treatment with 10 µM DAMGO, measured by surface receptor staining (n=5, two-tailed paired t-test, p=0.13).

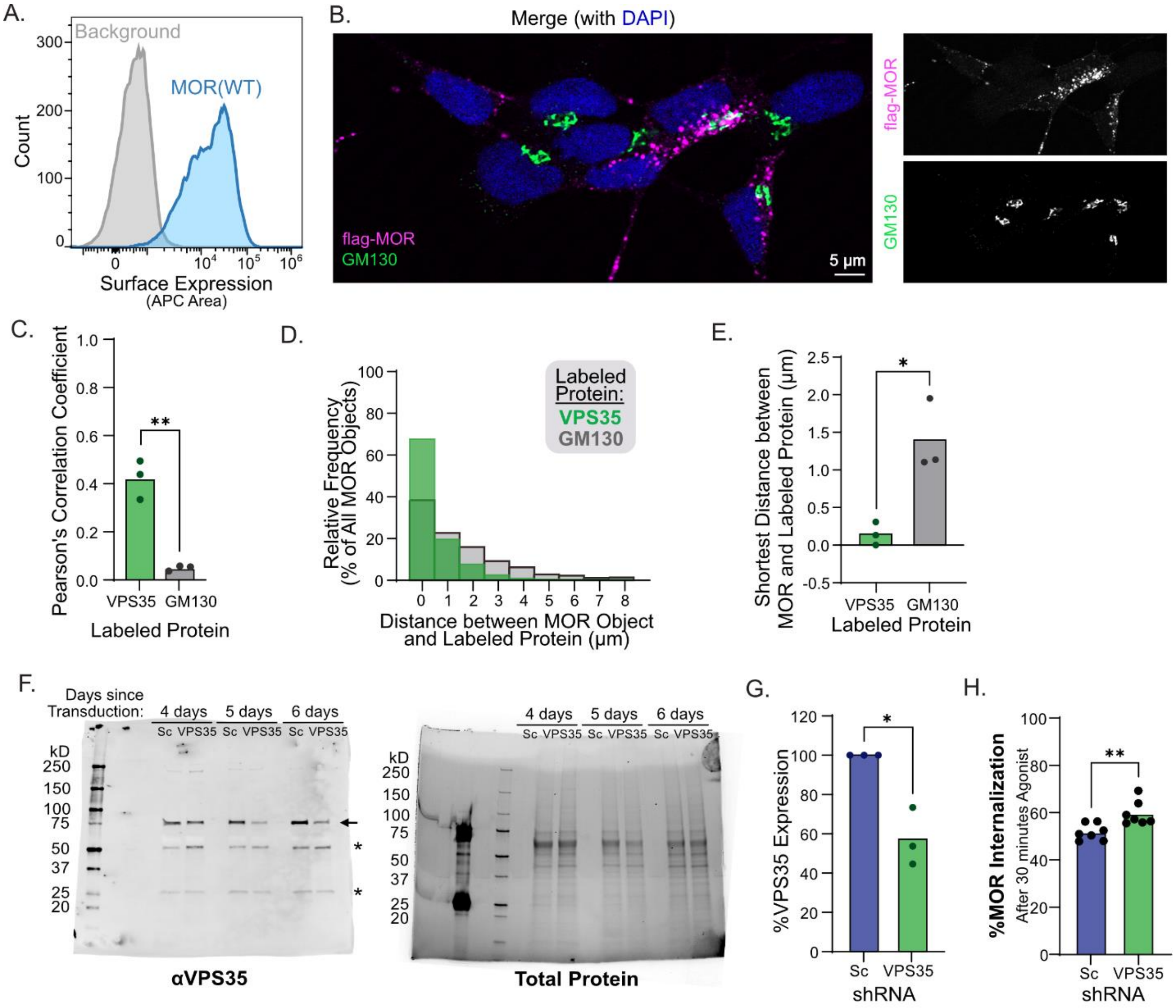

Fig. S5. Retromer's role in MOR recycling is conserved in SH-SY5Y cells. A. Flow cytometry histogram of MOR(WT) surface expression in SH-SY5Y lines. **B.** Example confocal image of SH-SY5Y cell stably expressing MOR(WT) labeled with anti-FLAG (magenta) and anti-GM130 (green). **C.** Pearson's correlation coefficient analysis for co-localization of MOR with VPS35 or GM130 (n=3, unpaired t-test, p=0.0014) in SH-SY5Y cells. **D.** Frequency distributions of the closest distance between an individual MOR(WT) object and a GM130 or VPS35 object in SH-SY5Y cells. **E.** Average closest distance between all MOR objects and a GM130 or VPS35 object in SH-SY5Y cells (n=3, two-tailed unpaired t-test, p=0.013). **F.** Western blot for VPS35 and total protein from SH-SY5Y lysates expressing MOR(WT) and either Sc or VPS35 shRNA. Three biological replicates are shown at different timepoints from transduction with shRNA. Arrow denotes VPS35. Asterisks denote off-target bands. **G.** Quantification of shRNA knock-down of VPS35 from 3 biological replicates at least five days after transduction, normalized for total protein and to Sc VPS35 expression (n=3, two-tailed paired t-test, p=0.037). **H.** Internalization of MOR(WT) in stably expressing SH-SY5Y cells

transduced with Sc or VPS35 shRNA and treated for 30 minutes with 10 μM DAMGO (n=7, two-tailed paired t-test, p=0.0058).

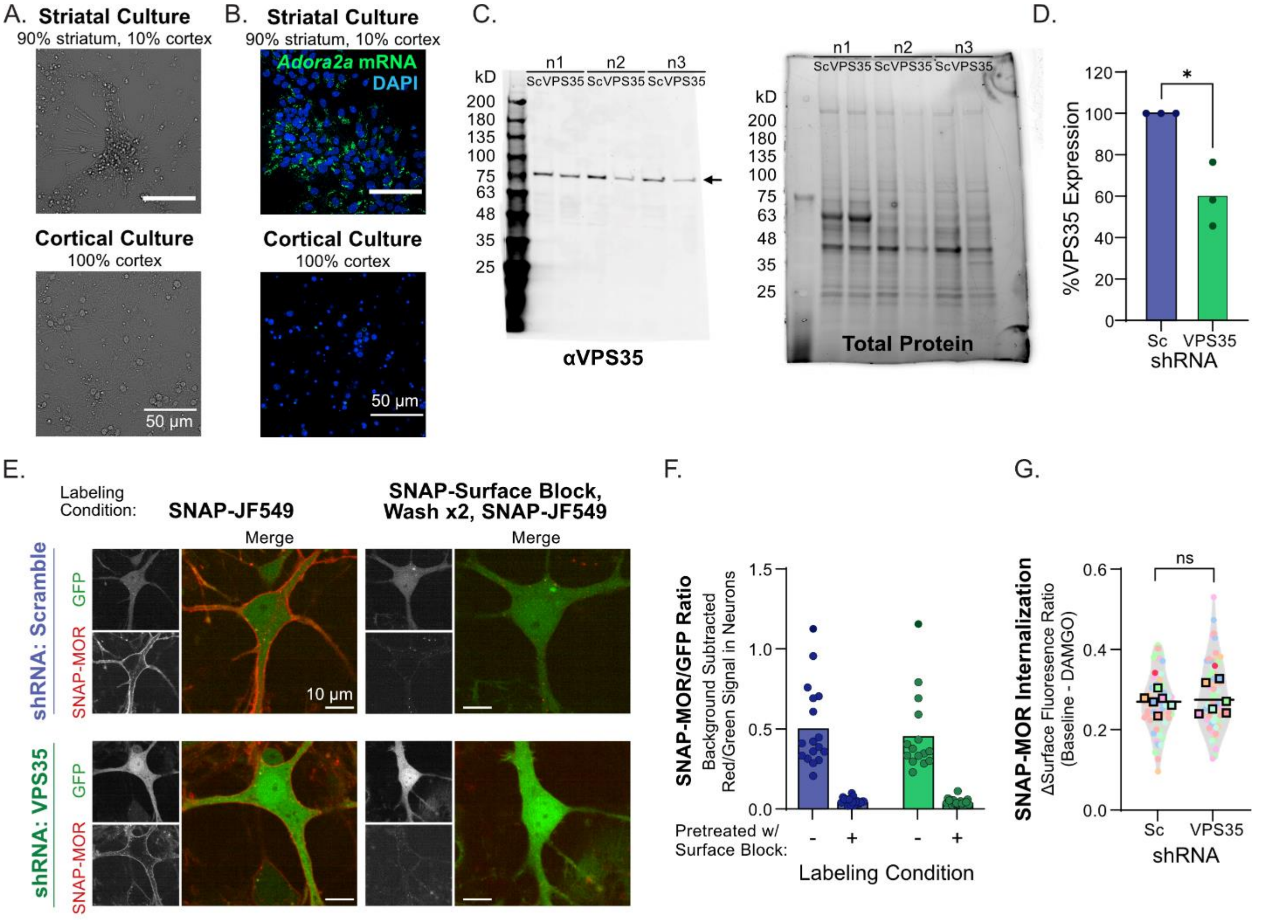

Fig. S6. Retromer's role in MOR recycling is conserved in rodent striatal neurons. A. Bright field images of neuronal cultures from striatal-cortical preparations (90% striatum / 10% cortex, top) and negative control pure cortical preparations (100% cortex, bottom). Representative images from an n=3 shown. **B.** Fluorescence in situ hybridization of Adora2a mRNA (green), a gene expression marker for spiny projection neurons, with DAPI nuclear staining (blue). Adora2a expression is largely restricted to striatal-cortical cultures (top) and not observed in negative control cortical cultures (bottom), consistent with selective enrichment of spiny projection neurons. Representative images from an n=3 shown. **C.** Western blot for VPS35 and total protein from murine striatal neuron culture lysates transduced with Sc or VPS35 shRNA/SNAP-MOR. Arrow denotes VPS35. All three biological replicates shown. **D.** Quantification of VPS35 expression in striatal neuronal cultures transduced with Sc or VPS35 shRNA/SNAP-MOR, normalized for total protein and to Sc VPS35 expression (n=3, two-tailed paired t-test, p=0.047). **E.** Confocal images of live murine striatal neurons transduced with Sc or VPS35 shRNA/SNAP-MOR, labeled with 1 µM SNAP-JF549 (red) for 15 minutes (left) or 1 µM SNAP-Surface Block for 15 minutes followed by 1 µM SNAP-JF549 for 15 minutes (right). GFP (transduction marker) is shown in green. Representative image shown from at least 15 cells per condition (n=17 for Sc no Block, n=17 for Sc block, n=15 for VPS35 no Block, n=18 for VPS35 Block). **F.** Quantification of the ratio of background-subtracted SNAP-MOR and GFP in striatal neurons described in Supplemental Figure E. Each point represents one neuron. **G.** Quantification of SNAP-MOR internalization in murine striatal neurons as the difference

between the surface fluorescence ratio at baseline and after 1 μM DAMGO for each individual neuron. Circles denote values for each individual neuron color-coded by their imaging session. Red circles denote the example neuron shown in Figure 5D. Squares denote the median value for each independent imaging session. Statistical analysis is performed on these median values (n=6, two-tailed paired t-test, p=0.82).

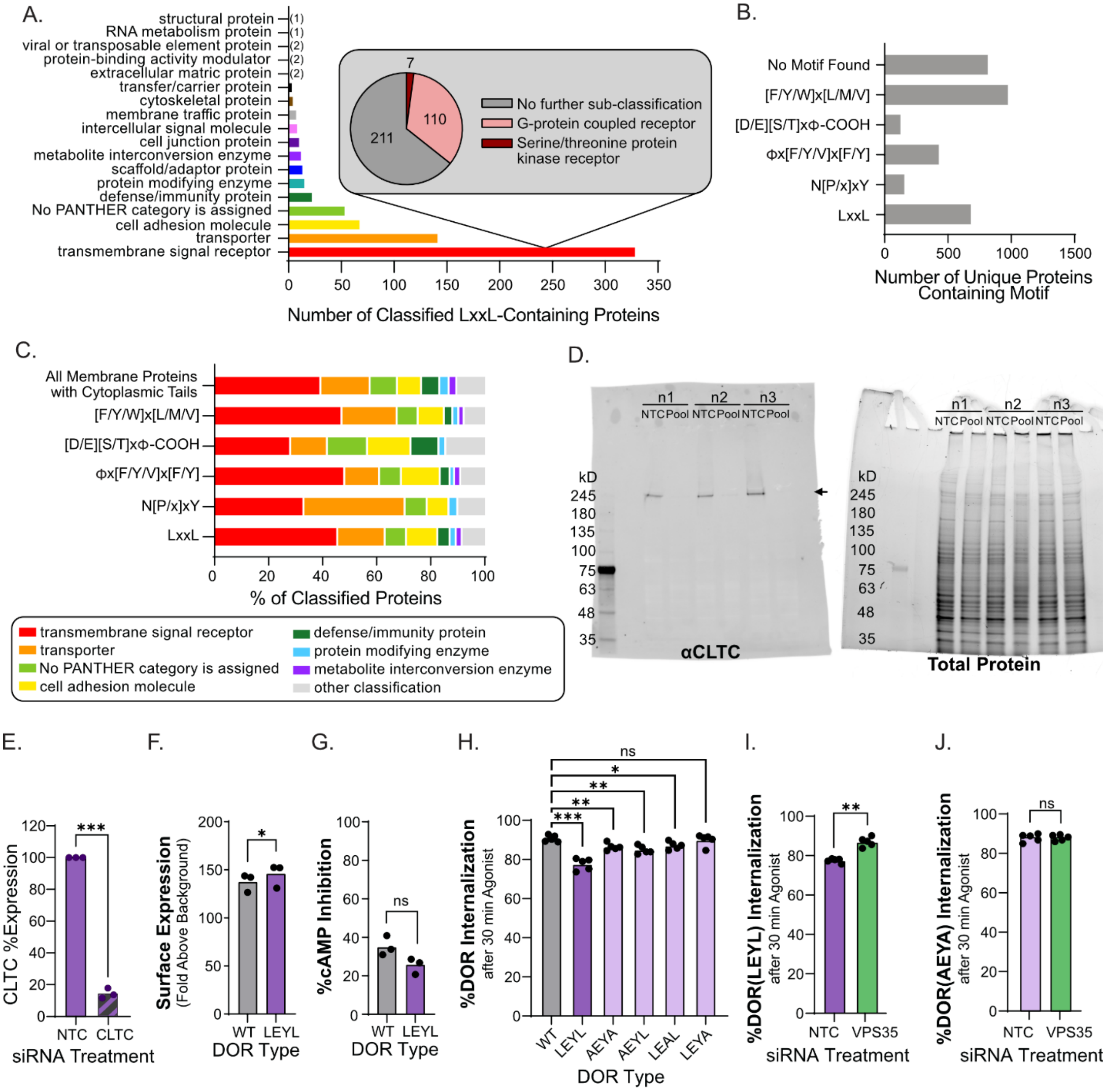

Fig. S7. Bileucine recycling motifs are a general mechanism for accessing Retromer-dependent recycling. A. PANTHER Protein Class analysis of membrane proteins with cytoplasmic-facing regions near the C-terminal tails containing "LxxL" motifs. **B.** Number of unique membrane proteins with cytoplasmic-facing regions near the cytoplasmic C-terminal tail containing each searched motif. **C.** PANTHER Protein Class analysis of membrane proteins with cytoplasmic-facing regions near the cytoplasmic C-terminal tail containing different recycling motifs. **D.** Western blot for CLTC and total protein from HEK293-TRex cell lysates expressing inducible GLUT4(WT) and treated with siRNAs against CLTC. Arrow denotes CLTC. All three biological replicates shown. **E.** Quantification of CLTC knock-down in HEK293-FLP-TRex cell lysates expressing inducible GLUT4(WT) and treated with siRNAs against CLTC, normalized to total protein and NTC CLTC levels (n=3, two-tailed paired t-test, p=0.00050). **F.** Surface

receptor expression detected by antibody labeling in HEK293-FLP cells stably expressing DOR(WT) or DOR(LEYL) compared to background antibody staining in a non-expressing parental line (n=3, two-tailed paired t-test, p= 0.043). DOR(WT) data are re-plotted from Supplemental Figure 1J – conditions were all performed on the same day but are plotted separately for clarity. **G.** Percent cAMP inhibition in HEK293-FLP cells transiently expressing DOR(WT) or DOR(LEYL) over 20 minutes of treatment with 10 μM DADLE and 30 nM isoproterenol (n=3, two-tailed paired t-test, p=0.22). **H.** Percent DOR internalization in HEK293-FLP cells stably expressing receptor following treatment with 10 μM DADLE for 30 minutes measured by surface receptor labeling (n=5, one-way RM ANOVA p=0.00010, 0.0046, 0.0015, 0.025, and 0.98 for DOR(WT) vs DOR(LEYL), DOR(AEYA), DOR(AEYL), DOR(LEAL), and DOR(LEYA) respectively). **I.** Percent DOR(LEYL) internalization in stably expressing HEK293-FLP cells following siRNA knock-down of VPS35 and treatment with 10 μM DADLE for 30 minutes measured by surface receptor labeling (n=5, two-tailed paired t-test, p=0.0012). **J.** Same as I, but in HEK293-FLP cells stably expressing DOR(AEYA) (n=5, two-tailed paired t-test, p=0.99).

Table S1.

Comparison of hits from genome-wide screen of DOR(WT) and DOR-MRS(WT).

Table S2.

Genome-wide screen hit gene expression analysis in neurons.

Table S3.

Bioinformatics search of recycling motifs in membrane proteins with cytoplasmic tails.

Table S4.

All data values presented in the main and supplemental figures.
